# Epilepsy: Molecular Pathogenesis and Emerging Therapies

**DOI:** 10.1002/mco2.70735

**Published:** 2026-04-12

**Authors:** Wanbin Huang, Jiabin Zong, Yu Zhang, Ming Li, Songqing Pan, Zheman Xiao

**Affiliations:** ^1^ Department of Neurology Renmin Hospital of Wuhan University Wuhan Hubei China; ^2^ Department of Encephalopathy in Traditional Chinese Medicine Renmin Hospital of Wuhan University Wuhan Hubei China; ^3^ Department of Neurology, Union Hospital, Tongji Medical College Huazhong University of Science and Technology Wuhan Hubei China

**Keywords:** clinical trials, effect evaluation, emerging therapy, epilepsy, molecular pathogenesis, precision medicine

## Abstract

Epilepsy is a prevalent and often severe neurological disorder with significant economic and societal impacts. Despite adequate trials with two or more antiseizure medications (ASMs), approximately one‐third of patients continue to experience drug‐resistant epilepsy. Surgery remains the most effective treatment for patients with drug‐resistant focal epilepsy, yet it may be underutilized. Given the unsatisfactory clinical outcomes, it is imperative to investigate the molecular pathogenesis of epilepsy and to develop novel therapeutic strategies. This review systematically outlines the advances in understanding the etiology and molecular pathogenesis of epilepsy. These advances have identified diverse therapeutic targets, stimulating the development of emerging therapies, including novel ASM, minimally invasive surgery, neurostimulation, focused ultrasound, nanomedicine, gamma‐aminobutyric acid (GABA)ergic cell therapy, and gene therapy. Additionally, the review comprehensively evaluates emerging therapies for epilepsy from different perspectives by integrating findings from both preclinical and clinical studies. These innovative approaches offer the potential for long‐term seizure control. A great deal of future research is still needed to overcome current shortcomings. This work provides a cohesive framework that bridges molecular mechanisms with therapeutic applications. Such efforts may provide novel ideas and optimizing approaches in the field, ultimately advancing the precision and effectiveness of epilepsy treatments in the future.

## Graphical abstract

Progress has been made in the molecular pathogenesis of epilepsy, revealing multiple therapeutic targets. Recent advances in pharmacology, materials science, and surgical technique, coupled with progress in targeted therapy and disruptive epilepsy network technology, have led to the emergence of innovative strategies for epilepsy treatment. These strategies encompass novel antiseizure medications, minimally invasive surgery, neural stimulation, nanomaterials, focused ultrasound, cell therapy, and gene therapy. They could substantially reshape existing treatment paradigms. The graphical abstract is drawn by Figdraw.

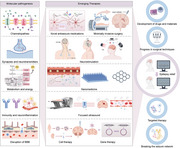

## 1 Introduction

Epilepsy is a neurological disorder characterized by abnormal excessive or synchronous neuronal activity in the brain [1]. Epileptic seizures manifest as paroxysmal episodes with transient motor, sensory, psychiatric, autonomic, and visual symptoms [2]. Epilepsy accounts for a significant portion of the global disease burden, affecting approximately 70 million individuals, and is associated with increased risks of physical harm and mortality, particularly in cases where treatment is inadequately managed [3]. Furthermore, 25%–50% of individuals with epilepsy also experience comorbid neurological, psychiatric, cognitive, or systemic disorders [4]. Therefore, revealing the potential development origin and mechanism basis of epilepsy and developing effective treatment strategies are important demands for epilepsy management.

Historically, the etiology and pathogenesis of epilepsy have been largely unknown. In recent years, our comprehension of the etiology and molecular pathogenesis of epilepsy has significantly advanced [5]. Progress in elucidating phenotypic patterns and underlying mechanisms has facilitated an update in the classification of etiology [6, 7]. This revised classification not only enhances diagnostic accuracy but also informs treatment strategies. Research into the mechanisms of epilepsy has expanded from a focus on simple neuronal hyperexcitability to encompass a complex array of interconnected mechanisms, including ion channel dysfunction, synaptic imbalance, immune responses, neuroinflammation, metabolic disorders, and disruption of the blood–brain barrier (BBB) [8, 9, 10, 11, 12, 13].

The field of epilepsy treatment has witnessed notable progress in the past decade. New antiseizure medications (ASMs), minimally invasive surgery, and neurostimulation have received clinical approval, offering alternative mechanisms of action that can enhance seizure control [14, 15, 16]. Focused ultrasound (FUS) can be employed for the ablation of the epileptogenic zone (EZ), neurostimulation, and the opening of the BBB [17]. As a noninvasive technique, FUS does not require craniotomy. When integrated with magnetic resonance imaging (MRI), FUS enables precise targeting with high spatial resolution [17]. Additionally, there is a growing emphasis on optimizing the delivery of ASMs. Preclinical studies in animal models suggest that nano‐delivery systems have the potential to improve the ability of ASMs to penetrate the BBB [18, 19]. Nanomaterials also enhance the efficacy and safety of neurostimulation, presenting an opportunity for integrating diagnosis and treatment [20]. Gamma‐aminobutyric acid (GABA)ergic cell therapy enhances inhibitory neurotransmission and mitigates the neuronal hyperexcitability [21]. Gene therapy provides a more precise intervention by directly addressing the genetic and molecular causes of epilepsy [22]. Many patients are expected to benefit from these emerging therapies. Ongoing research aims to further refine these approaches, steering the field toward precise and personalized therapies.

The understanding of the molecular biological mechanisms and intervention strategies for epilepsy is continually evolving and expanding. However, existing literature reviews in this domain remain insufficiently comprehensive and systematic, as they lack multidimensional mechanism sorting, evaluation of emerging therapies, integration of preclinical and clinical evidence, comparison with traditional treatments, and analysis of clinical translation pathways. Consequently, it is necessary to conduct a detailed and thorough review of the etiology, molecular pathogenesis, and emerging therapies for epilepsy. This review aims to elucidate critical unresolved issues and propose potential solutions, thereby encouraging further research and translational applications in this field. These efforts hold promise for advancing the precision and effectiveness of epilepsy therapy in the future.

In this review, we initially outline the etiology of epilepsy, establishing a foundation for comprehending the origins of the disease. Subsequently, we explore the principal aspects of molecular pathogenesis. The following section provides an in‐depth overview of innovative epilepsy therapies. We offer a critical evaluation of findings derived from preclinical and clinical studies to highlight the potential advantages and limitations of emerging therapies. By following a structured progression from etiology to molecular pathogenesis and subsequently to emerging therapies, this review presents a comprehensive and coherent analysis of the current landscape and future directions in epilepsy management.

## 2 Etiology of Epilepsy

**FIGURE 1:**
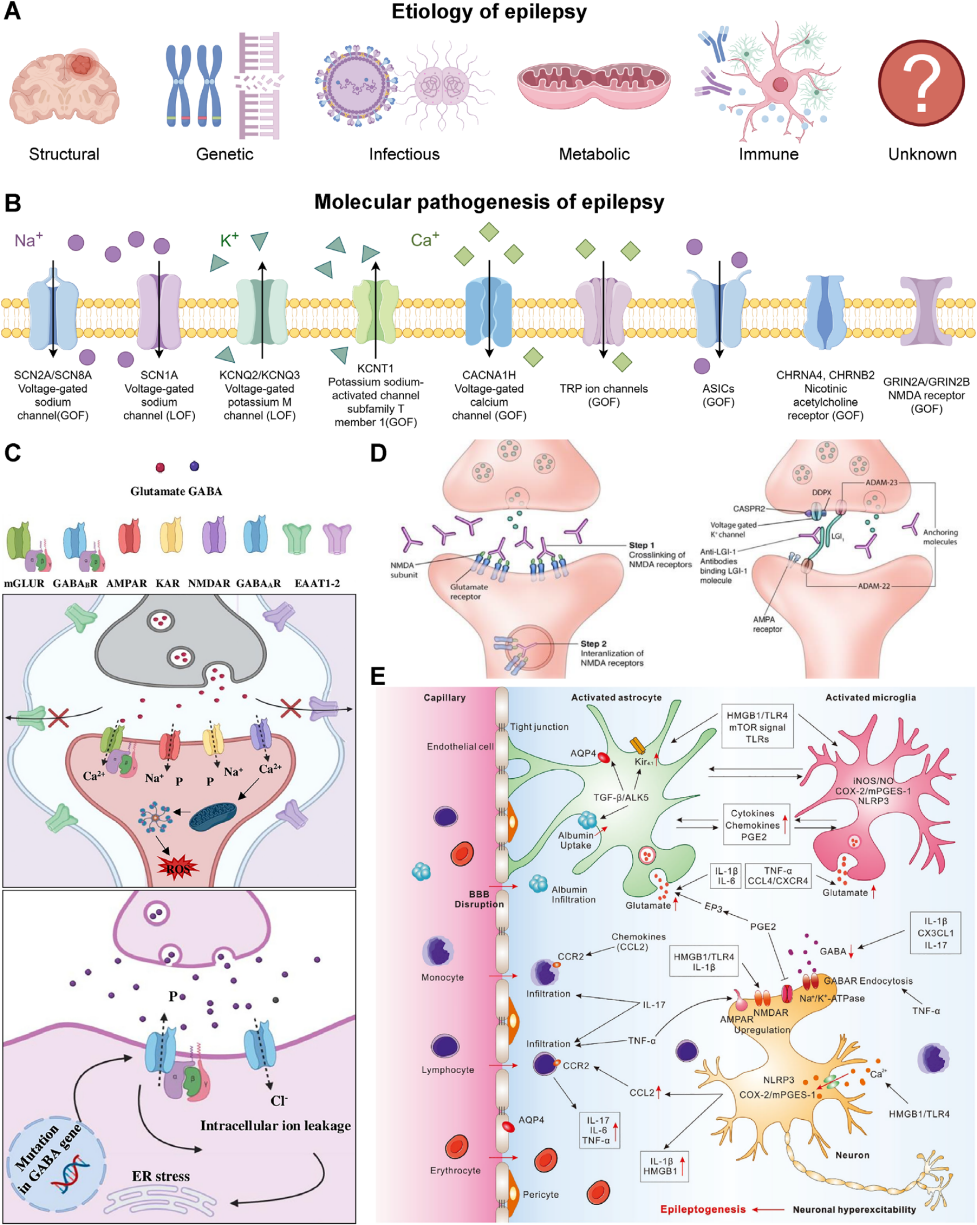
Etiology and molecular pathogenesis of epilepsy. (A) Etiology of epilepsy is divided into six distinct categories: structural, genetic, infectious, metabolic, immune, and unknown. The figure is drawn by Figdraw. (B) Channelopathies in epilepsy. Adapted with permission from Ref. [23]. Copyright 2021 American Academy of Neurology. The figure is drawn by Figdraw. (C) Enhanced activity of NMDAR and AMPAR elevates intracellular Ca^2+^ levels, promoting ROS generation. Elevated glutamate levels contribute to neuronal excitotoxicity. Subsequently, oxidative stress induces apoptosis through mitochondrial dysfunction. Additionally, mutations in GABA receptor subunit genes alter GABAAR function, leading to impaired Cl^−^ influx and triggering processes such as intracellular ion imbalance and endoplasmic reticulum stress. Adapted with permission from Ref. [24]. Copyright 2021 Elsevier. (D) Anti‐NMDAR antibodies target the extracellular domain of NMDARs, impairing receptor surface density through cross‐linking and internalization. Anti‐LGI1 antibodies disrupt the interaction between LGI1 and ADAM22/23, thereby indirectly compromising AMPAR function. Adapted with permission from Ref. [25]. Copyright 2022 MDPI. (E) Neuroinflammation and BBB disruption are involved in epilepsy pathogenesis. Reprinted with permission from Ref. [26]. Copyright 2023 Frontiers. BBB, blood–brain barrier; GABA, gamma‐aminobutyric acid; GOF, gain‐of‐function; HMGB1, high mobility group box 1; IL‐1β, interleukin‐1β; LOF, loss‐of‐function; NMDAR, *N*‐methyl‐d‐aspartate receptor; TGF‐β, transforming growth factor‐beta; TNF‐α, tumor necrosis factor‐α; TRP, transient receptor potential.

The most authoritative international classification system for epilepsy etiology is proposed by the International League Against Epilepsy. The most recent classification framework was introduced in the 2017 guidelines and continues to be emphasized in subsequent updates [6, 7]. The etiology of epilepsy is divided into six distinct categories: structural, genetic, infectious, metabolic, immune, and unknown (Figure 1A). These categories provide a framework for understanding disease origins.

Structural etiology is defined by neuroimaging abnormalities that significantly correlate with an increased risk of epilepsy. These abnormalities can be acquired (such as traumatic brain injury) or genetic (such as focal cortical dysplasia) [6]. The detection of a structural lesion often guides evaluation for epilepsy surgery. Notably, a structural lesion may have a genetic basis. An example is tuberous sclerosis complex (TSC), in which mutations in the *TSC1* or *TSC2* genes cause structural changes [27].

A genetic etiology implies the epilepsy is directly caused by a known or presumed genetic variant, with seizures as a core symptom. This category includes several types: (1) monogenic disorders, such as sodium voltage‐gated channel alpha subunit 1 (*SCN1A*) mutations in Dravet syndrome (DS) and potassium voltage‐gated channel subfamily Q member 2 (*KCNQ2*) mutations in neonatal epilepsies [28, 29, 30]; (2) polygenic inheritance, where multiple genetic variants contribute to susceptibility, often without a strong family history [31, 32]; (3) de novo mutations, which are frequently identified in developmental and epileptic encephalopathy (DEE) and are not inherited from the parents [29]; and (4) somatic mosaicism, which can affect phenotypic severity [33].

An infectious etiology is identified when epilepsy results directly from a central nervous system (CNS) infection. Globally, it is a major factor contributing to epilepsy. Examples include neurocysticercosis, cerebral malaria, acquired immunodeficiency syndrome, and congenital infections [6]. Seizures may occur during active infection or later as a sequela, such as postencephalitic epilepsy [34].

Metabolic epilepsies arise from well‐defined metabolic disturbances in which seizures are a core feature. These disorders often have a genetic basis (e.g., pyridoxine‐dependent epilepsy, porphyria, and aminoacidopathies) but can sometimes be acquired (e.g., cerebral folate deficiency) [6]. Early diagnosis is essential because targeted metabolic interventions may control seizures and prevent neurodevelopmental impairment [35].

Immune etiology refers to epilepsy caused by immune‐mediated inflammation in the CNS. The diagnosis of these disorders is supported by the identification of specific neural autoantibodies, such as anti‐N‐methyl‐D‐aspartate receptor (NMDAR) and anti‐leucine‐rich glioma inactivated 1 (LGI1) antibodies. Such conditions are increasingly acknowledged in both pediatric and adult patients. They have important treatment implications, as these patients often respond to immunotherapy [34, 36].

Etiology is classified as unknown when no cause can be identified with available diagnostic methods. The chance of finding an etiology depends on the extent of evaluation, which varies across healthcare settings. This category underscores the need for continued investigation since the diagnostic technologies advance [6].

The six etiological categories are not mutually exclusive. For example, genetic mutations can increase susceptibility to structural defects, and metabolic disturbances may exacerbate immune reactions [6]. Their interplay and molecular basis remain critical for elucidating pathogenesis. In the following chapter, we will introduce the molecular mechanisms that translate these etiological drivers into neuronal hyperexcitability.

## 3 Molecular Pathogenesis of Epilepsy

The molecular pathogenesis of epilepsy constitutes a complex domain. It results from an imbalance between neuronal excitatory and inhibitory signals at the molecular level [37]. This imbalance arises from multiple mechanisms. Key factors include dysfunction of ion channels, impaired synaptic transmission, immunity, neuroinflammation, metabolic disturbances, and disruption of the BBB. The following sections detail these core mechanisms.

### 3.1 Ion Channel Dysfunction

Epilepsy often arises from the dysregulation of ion channels that control neuronal excitability. These ion channels mainly include voltage‐gated sodium, potassium, and calcium channels, transient receptor potential (TRP) channels, acid‐sensing ion channels (ASICs), hyperpolarization‐activated cyclic nucleotide‐gated (HCN) channels, and ligand‐gated ion channels (LGICs) such as GABA receptors [8, 38, 39, 40, 41] (Figure 1B). Mutations or functional alterations in these channels may induce hyperexcitability, synchronization of neuronal activity, and contribute to the process of epileptogenesis.

Voltage‐gated sodium channels are essential for initiating and propagating action potentials. Gain‐of‐function mutations in these channels can slow inactivation or increase the open probability of sodium channels. This enhances neuronal excitability and promotes high‐frequency discharges [42]. For instance, gain‐of‐function mutations in sodium voltage‐gated channel alpha subunit 2 are associated with benign familial neonatal epilepsy (BFNE) [8]. Similarly, gain‐of‐function mutations in sodium voltage‐gated channel alpha subunit 8 elevate persistent sodium currents, leading to hyperexcitability and severe epileptic encephalopathy [43]. Conversely, loss‐of‐function mutations can also induce seizures by diminishing inhibitory neurotransmission. For instance, the *SCN1A* gene encodes the voltage‐gated sodium channel subtype 1.1 (Nav1.1), predominantly expressed in GABAergic interneurons. Loss‐of‐function mutations in *SCN1A* compromise the firing of inhibitory neurons, leading to network hyperexcitability and DS [8].

Potassium channels play an important role in regulating neuronal repolarization and excitability. Dysfunction in potassium channels can increase susceptibility to epilepsy. For example, mutations in the potassium voltage‐gated channel subfamily A member 1 result in a loss‐of‐function of potassium channels, impairing repolarization and leading to conditions such as episodic ataxia and epilepsy [44]. Similarly, mutations in the *KCNQ2* and potassium voltage‐gated channel subfamily Q member 3 diminish the M‐type potassium current. This reduction elevates neuronal excitability and action potential firing, leading to BFNE [44]. Furthermore, gain‐of‐function mutations in the potassium sodium‐activated channel subfamily T member 1 have been associated with autosomal dominant nocturnal frontal lobe epilepsy (ADNFLE). These mutations enhance sodium‐activated potassium currents, thereby disrupting neuronal firing patterns [45].

Voltage‐gated calcium channels are integral to modulating neurotransmitter release and neuronal excitability. Mutations in calcium voltage‐gated channel subunit alpha1 A can alter calcium influx, leading to familial hemiplegic migraine and epilepsy by altering calcium influx [46]. In childhood absence epilepsy, gain‐of‐function mutations in calcium voltage‐gated channel subunit alpha1 H increase T‐type calcium currents in thalamic neurons. This change promotes burst firing and spike‐wave discharges [46]. Additionally, dysregulation of voltage‐gated calcium channel type 2.3 (Cav2.3) has been implicated in epilepsy. For instance, in cyclin‐dependent kinase‐like 5 deficiency disorder, enhanced Cav2.3‐mediated calcium entry may lead to excitotoxicity [47].

TRP ion channels are tetrameric, nonselective cation channels. Several calcium‐permeable TRP channels play roles in intracellular calcium homeostasis and have been linked to acute seizures and chronic epilepsy [48]. These channels are typically expressed at basal levels under normal physiological conditions but calcium voltage‐gated channel be significantly upregulated in the epileptic brain, particularly within epileptic foci [38]. For example, transient receptor potential canonical 3 (*TRPC3*) channels are upregulated in temporal lobe epilepsy (TLE) and status epilepticus (SE) models. This upregulation enhances calcium influx and neuronal hyperexcitability. Pharmacological inhibition of TRPC3 (using agents such as pyrazole‐3 or JW‐65) has been shown to reduce seizure severity and duration [48].

ASICs respond to changes in extracellular pH and contribute to excitotoxicity. ASIC1a activation under acidic conditions leads to substantial Na^+^ and Ca^2^^+^ influx, promoting the release of neurotransmitters and potentially causing neuronal damage [39]. In TLE, ASIC1a upregulation in reactive astrocytes is associated with calcium overload and epileptogenesis. Inhibiting ASIC1a reduces seizure activity [49]. Additionally, ASIC1a also interacts with NMDAR and calcium/calmodulin‐dependent protein kinase II, exacerbating excitotoxicity and changes in synaptic plasticity that favor hyperexcitability [39].

HCN channels modulate neuronal excitability through Ih currents. In various models of acquired epilepsy, such as febrile seizures, pilocarpine‐induced SE, and cortical dysplasia, a prevalent alteration is the persistent downregulation of *HCN1* subtype mRNA and protein expression in the pyramidal neurons of the hippocampal CA1 region [40]. This downregulation results in a marked reduction in dendritic Ih current amplitude, thereby enhancing dendritic excitability and predisposing to seizures [50].

LGICs also play a critical role in epilepsy pathogenesis. Functional abnormalities in LGICs such as GABAA receptors (GABAAR), NMDAR, and nicotinic acetylcholine receptors (nAChR) are closely associated with the onset and progression of epilepsy [41, 51]. For example, loss‐of‐function mutations in GABAAR subunits impair inhibitory transmission, resulting in generalized epilepsy and epileptic encephalopathies [41]. Mutations in NMDAR genes may cause either gain‐ or loss‐of‐function effect, altering excitatory synaptic transmission and contributing to epileptic encephalopathy [51]. Similarly, mutations in nAChRs often enhance receptor function or increase sensitivity to acetylcholine, leading to abnormal cholinergic excitation and ADNFLE [51].

### 3.2 Synaptic Transmission and Neurotransmitter Imbalance

Research has shown that there are large‐scale gene expression changes in the hippocampus of patients with TLE, some of which are related to synaptic and neurotransmitter transmission [9] (Figure 1C). This disruption often involves structural and functional changes at synapses, alongside altered levels of key neurotransmitters. In this section, we primarily discuss how the dysregulation of synaptic transmission and neurotransmitters mediates the imbalance between excitation and inhibition in the epileptic brain.

In patients with epilepsy, a series of pathological alterations occur at synapses. One notable synaptic change is mossy fiber sprouting in the hippocampus, which is commonly observed in TLE [52]. Studies have shown that mossy fibers not only sprout within the dentate gyrus but also extend into the CA2 region, where they undergo synaptic reorganization. This process leads to the formation of abnormally large synaptic boutons that envelop CA2 pyramidal neurons. Such structural changes are likely to modify synaptic transmission and facilitate epileptic activity [53]. Synaptic plasticity is also recognized as a key factor in the pathological progression of TLE. Proteomic analyses have revealed a significant upregulation of synaptic plasticity‐related proteins in the hippocampus of epileptic rats, indicating that synaptic remodeling may contribute importantly to the pathogenesis of TLE [54]. These synaptic alterations in both structure and function can result in hyperexcitability of neuronal circuits, thereby promoting the generation of epileptic seizures.

Glutamate serves as the primary excitatory neurotransmitter in the CNS. Excessive accumulation of extracellular glutamate is associated with the initiation and maintenance of seizures [24]. One underlying mechanism involves alterations in the glutamate/glutamine cycle (GGC). During the early phase of epilepsy, glutamate turnover in the GGC is enhanced, resulting in increased glutamate supply [55]. In chronic epilepsy, astrocyte proliferation and neuronal loss lead to further disruptions in the GGC. These include reduced expression of glutamine synthetase and the astrocytic glutamate transporter‐1, which impairs glutamate clearance [55] (Figure 1C). Concurrently, neuronal phosphate‐activated glutaminase is activated, and the neuronal glutamate transporter shows increased activity. These changes promote glutamate release and elevate extracellular glutamate concentrations [55].

GABA is the major inhibitory neurotransmitter in the CNS. GABAergic neurotransmission is disrupted in patients with epilepsy [56]. A key mechanism involves reduced GABA release and increased GABA reuptake. Consequently, neuronal inhibition is weakened, resulting in a relative increase in excitability that can trigger seizures [56]. For example, glutamate decarboxylase (GAD) catalyzes the synthesis of GABA from glutamate. Reduced GAD activity causes insufficient GABA production, contributing to epilepsy [57]. Additionally, increased activity of GABA transaminase (GABA‐AT) accelerates GABA degradation. This leads to decreased GABA levels in the brain, further promoting seizures. Inhibiting GABA‐AT can raise GABA levels and help terminate seizures [58].

### 3.3 Immunity and Neuroinflammation

The interplay between inflammation and immune mechanisms is integral to epileptogenesis. Innate immune responses promote neuroinflammation, whereas adaptive immune mechanisms can lead to autoimmune‐related neuronal dysfunction [10, 26]. This section describes these processes based on current evidence, highlighting key molecular pathways and cellular actors.

Immune mechanisms in epilepsy involve autoantibodies targeting neuronal antigens and T‐cell‐mediated cytotoxicity, often resulting in autoimmune encephalitis and chronic epilepsy. Under pathological conditions, B cells and plasma cells generate autoantibodies against neuronal surface proteins. These antibodies enter the CNS and disrupt synaptic function. Examples include antibodies against NMDAR, LGI1, and GABAAR [59]. Anti‐NMDAR antibodies trigger receptor internalization, leading to a reduction in synaptic NMDA currents and impairment of synaptic plasticity, which manifest clinically as seizures and psychiatric symptom [59] (Figure 1D). Anti‐LGI1 antibodies interfere with the binding between LGI1 and a disintegrin and metalloproteinase 22/23 (ADAM22/23), resulting in reduced clustering of α‐amino‐3‐hydroxy‐5‐methyl‐4‐isoxazolepropionic acid (AMPA) receptors and faciobrachial dystonic seizures [59] (Figure 1D). Anti‐GABAAR antibodies directly inhibit GABAAR function, diminishing inhibitory neurotransmission and causing severe refractory seizures [59]. T‐cell cytotoxicity also contributes to immune‐mediated epilepsy. In paraneoplastic syndromes and anti‐GAD encephalitis, CD8+ T cells infiltrate the CNS. These cells release granzyme B and perforin, inducing neuronal apoptosis [10]. Additionally, T cells secrete cytokines such as interferon‐γ (IFN‐γ), which activate microglia and enhance neuroinflammation [10].

Inflammation contributes to epilepsy mainly by activating innate immune cells and releasing pro‐inflammatory mediators, which increase neuronal excitability and support epileptogenesis (Figure 1E). Seizures trigger the activation of microglia and astrocytes. These cells then produce pro‐inflammatory cytokines, including interleukin‐1β (IL‐1β), tumor necrosis factor‐α (TNF‐α), and IL‐6 [26]. IL‐1β enhances neuronal excitability by increasing glutamate release and reducing GABAergic inhibition. This occurs through IL‐1 receptor type 1 signaling, which phosphorylates NMDAR via Src kinase. This phosphorylation results in elevated calcium influx and hyperexcitability [60]. TNF‐α disturbs the balance between excitation and inhibition by increasing AMPA receptor expression and promoting GABA receptor endocytosis [26]. Activated glial cells also release cytokines and chemokines, which attract peripheral immune cells and sustain inflammation [60].

Neuroinflammation also drives epilepsy through other mechanisms, such as inflammasome activation and prostaglandin pathways. The NOD‐like receptor family pyrin domain containing 3 is activated by damage‐associated molecular patterns, such as adenosine triphosphate (ATP) and high mobility group box 1 (HMGB1). This activation cleaves pro‐IL‐1β into its active form via caspase‐1 [11]. HMGB1 released during seizures from neurons and glia binds to Toll‐like receptor 4. This binding triggers nuclear factor kappa B signaling and promotes pro‐inflammatory gene expression [60]. After seizures, cyclooxygenase‐2 expression rises, converting arachidonic acid to prostaglandin E2 (PGE2). PGE2 then acts on PGE2 receptors to alter synaptic transmission and intensify inflammation [61].

### 3.4 Metabolic and Energy Disorders

Energy and metabolic disturbances are key components in the molecular pathology of epilepsy [12]. The brain requires substantial energy, mainly supplied by glucose oxidation and mitochondrial ATP generation. In epilepsy, these energy supply mechanisms are impaired, leading to metabolic imbalances that the initiation and spread of seizures [12]. Studies of epilepsy patients and animal models show notable metabolite alterations, such as elevated lactate, glutamate, and ketone bodies, along with decreased citrate and glucose utilization. These changes reflect a shift toward glycolytic metabolism and weakened mitochondrial function [6].

Glycogen, predominantly stored in astrocytes, functions as a critical energy reserve during seizure events. Notably, interictal glycogen levels are elevated in epileptogenic regions, despite a decrease in glucose utilization as evidenced by 18‐fluorodeoxyglucose positron emission tomography (FDG‐PET), indicating the presence of compensatory mechanisms [62]. Glycogenolysis supplies astrocytes with energy, thereby allowing a greater availability of blood‐derived glucose for neuronal use. However, excessive depletion of glycogen may lead to energy deficits in astrocytes, potentially impairing their potassium buffering capacity and contributing to the pathogenesis of seizures [62].

Mitochondria play a central role in cellular energy production. Mitochondrial dysfunction in epilepsy leads to reduced ATP synthesis, increased reactive oxygen species (ROS), and oxidative damage to proteins, lipids, and deoxyribonucleic acid (DNA). These pathological changes exacerbate neuronal hyperexcitability and cell death [63]. Mitochondrial disorders, such as myoclonic epilepsy with ragged‐red fibers, frequently arise from mutations in mitochondrial DNA, leading to impaired electron transport chain function and increased oxidative stress [64]. In this context, microglia and astrocytes produce ROS and reactive nitrogen species through the activation of nicotinamide adenine dinucleotide phosphate oxidase. These molecules disrupt ionic homeostasis and promote hyperexcitability. Moreover, oxidative stress facilitates the release of HMGB1, thereby exacerbating inflammation [26].

Furthermore, inborn errors of metabolism, such as pyridoxine‐dependent epilepsy and serine biosynthesis defects, directly cause epilepsy by interfering with neurotransmitter production, the transport of energy substrates, or redox balance [65]. These disorders commonly present with early‐onset seizures, developmental delays, and distinctive metabolic profiles, highlighting the important role of metabolic stability in controlling neuronal excitability [65].

### 3.5 Disruption of the BBB

The BBB is a critical interface that maintains brain homeostasis by controlling the movement of molecules and cells between the blood and brain parenchyma [66]. BBB disruption contributes to epilepsy through multiple pathways, including the entry of peripheral substances, neurovascular unit dysfunction, and drug resistance mechanisms [13]. Seizures can damage the BBB, creating a cycle where seizure activity further impairs barrier function through glutamate‐triggered matrix metalloproteinase (MMP) activation and inflammation [66]. This cycle worsens the progression of epilepsy.

BBB disruption allows for the extravasation of serum albumin into the brain parenchyma (Figure 1E). Astrocytes take up albumin, activating transforming growth factor‐beta (TGF‐β) signaling. This leads to reduced expression of potassium channels and GLTs, thereby impairing potassium buffering and increasing neuronal hyperexcitability [13]. Albumin‐induced TGF‐β signaling also causes astrocytic inflammation and synaptic reorganization, further supporting epileptiform activity [67]. In addition, BBB disruption allows peripheral immune cells (e.g., leukocytes) to enter the brain and release pro‐inflammatory cytokines. These cytokines intensify neuroinflammation and lower the seizure threshold [68]. Activated microglia and astrocytes produce MMPs, which degrade tight junction proteins (e.g., zonula occludens‐1) and further damage BBB integrity [69].

The dysfunction of the BBB is linked to angiogenesis and vascular remodeling in epilepsy. Increased vascular endothelial growth factor signaling raises vascular permeability and contributes to epileptogenesis through neurovascular unit impairment [70]. In post‐traumatic epilepsy and poststroke epilepsy, BBB disruption after injury or ischemia leads to hemorrhagic transformation, vasogenic edema, and leakage of excitotoxic molecules (e.g., iron and thrombin), initiating and sustaining seizures [71].

Seizures or injury can also upregulate ATP‐binding cassette transporters, such as P‐glycoprotein and breast cancer resistance protein, at the BBB. These transporters reduce the brain uptake of ASMs, leading to pharmacoresistance [67]. This transporter hypothesis proposes that efflux pump overexpression limits drug delivery to epileptogenic areas, maintaining seizure activity [67].

The molecular pathogenesis of epilepsy does not arise from isolated mechanisms. Instead, it involves a dynamic network of synergistic interactions among multiple pathways. For instance, upregulation of the TRPC3 channel in TLE exacerbates calcium overload. Simultaneously, mossy fiber sprouting and neuroinflammation further amplify this pathological cascade [72, 73, 74]. This comprehensive understanding reveals potential therapeutic targets, providing a novel perspective for the development of epilepsy treatment strategies.

## 4 Emerging Therapies of Epilepsy

Up to now, epilepsy treatment is still based on ASMs, with the primary goal of eliminating epileptic seizures [4]. The unsatisfactory clinical outcomes highlight the urgent need for novel therapeutic strategies that can offer better efficacy, safety, and precision [75]. Despite advances in understanding epilepsy mechanisms, therapeutic challenges persist. Emerging therapies extend beyond conventional ASMs to include surgical innovations, neurostimulation, FUS, nanomedicine, and novel biologic interventions. This section examines these advanced approaches, focusing on their mechanisms, clinical efficacy, and potential to address the limitations of current treatments.

### 4.1 Advances in ASMs

Pharmacotherapy remains the cornerstone of epilepsy treatment. Despite the introduction of numerous new ASMs over the past two decades, the prevalence of drug‐resistant epilepsy (DRE) remains unchanged, underscoring the urgent need for innovative therapies [75]. Recently, several novel ASMs have been approved for DRE, offering good efficacy, fewer adverse effects, and reduced drug resistance [76]. These agents have also shown promise as adjunctive therapies for DEE. Additionally, new formulations of intranasal benzodiazepines represent a significant advance in rescue therapy, effectively controlling acute repetitive seizures [14]. Multiple clinical studies have demonstrated their clinical application value.

#### 4.1.1 Novel Approved ASMs

**FIGURE 2:**
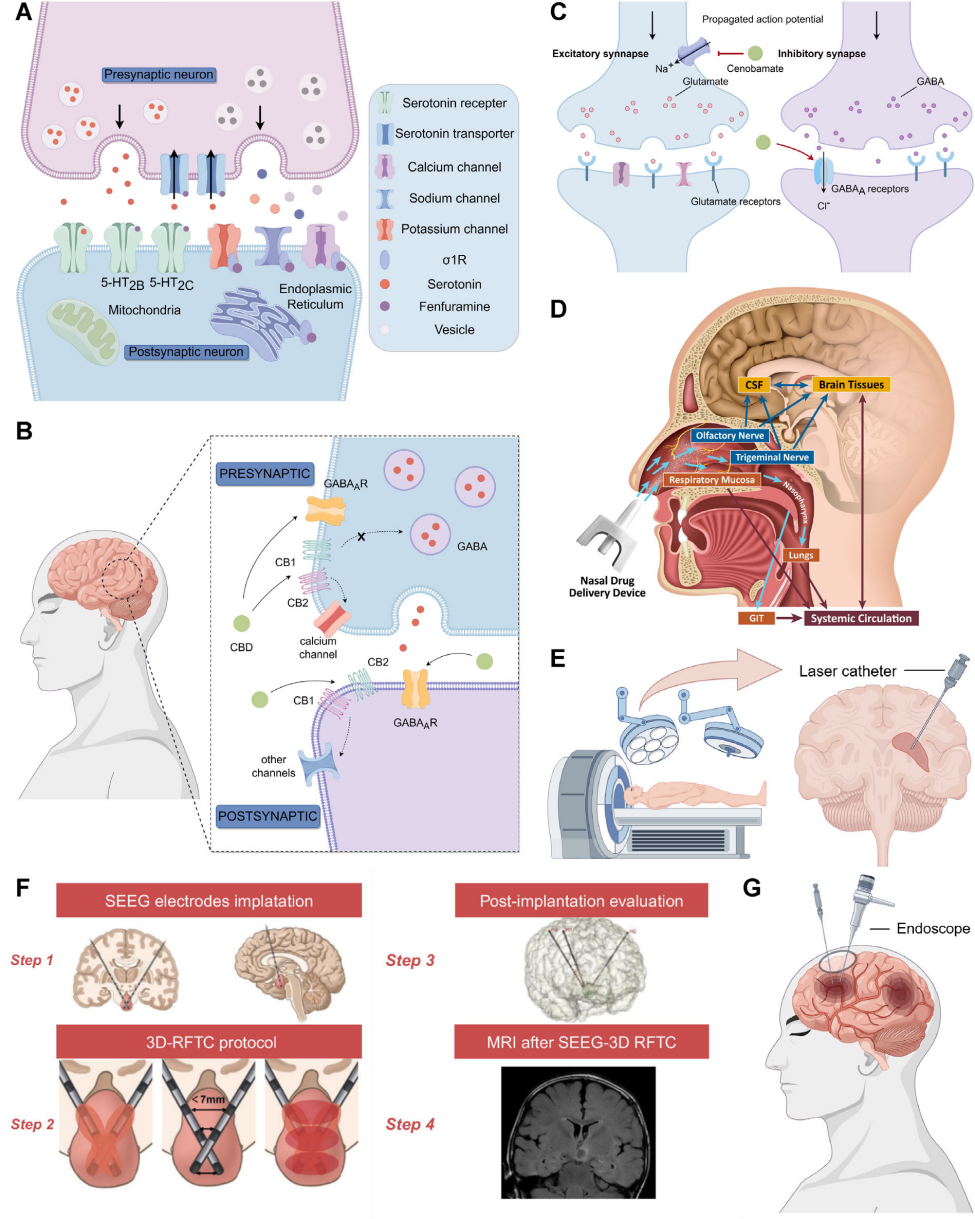
Novel ASMs and minimally invasive surgery in epilepsy treatment. (A) FFA induces serotonin release through disruption of vesicular storage, reversal of serotonin transporter function, and activation of specific serotonin receptors. In addition, FFA regulates a variety of ion channels and mitochondrial‐associated endoplasmic reticulum via sigma‐1 receptor stimulation. The figure is drawn by Figdraw. (B) CBD can modulate GABAAR and enhance GABA function by interacting with cannabinoid receptor 1 (CB1) and cannabinoid receptor 2 (CB2). Adapted with permission from Ref. [78]. Copyright 2021 MDPI. The image is drawn by Figdraw. (C) Cenobamate acts as a positive allosteric modulator of GABAAR, consistently blocking sodium channel currents and enhancing GABAergic signaling. The image was drawn by Figdraw. (D) Intranasal drug delivery routes: direct to the brain or through systemic absorption. Reprinted with permission from Ref. [99]. Copyright 2021 John Wiley & Sons. (E) Schematic diagram of LITT. The image was drawn by Figdraw. (F) Schematic diagram of SEEG‐guided RFTC. Adapted with permission from Ref. [100]. Copyright 2025 John Wiley & Sons. (G) Schematic diagram of endoscopic surgery. The image was drawn by Figdraw. CBD, cannabidiol; CSF, cerebrospinal fluid; GABA, gamma‐aminobutyric acid; GIT, gastrointestinal tract; RFTC, radiofrequency thermocoagulation; SEEG, stereoelectroencephalography.

Current ASM development prioritizes efficacy and tolerability. We focus on three emerging ASMs: fenfluramine (FFA), cannabidiol (CBD), and cenobamate (CNB), with their pharmacological properties detailed in Table 1. FFA acts as an agonist on 5‐HT2 and sigma‐1 receptors, promoting serotonin and GABA transmission while inhibiting glutamatergic activity [77] (Figure 2A). Approval was granted to FFA for the management of DS in 2020 and Lennox Gastaut syndrome (LGS) in 2022 [76]. In recent years, studies have substantiated the effectiveness of CBD in epilepsy management, acting through mechanisms such as modulation of ionic channels and neurotransmitter transporters to suppress seizures [78] (Figure 2B). CBD has received approval for the treatment of focal seizures associated with DS, LGS, and TSC. CNB blocks sodium channel currents and enhances GABAergic activity by serving as a positive allosteric modulator of GABAAR [79] (Figure 2C). CNB was approved for the treatment of adults with focal seizures around 2020 [6].

**TABLE 1:** The characteristics of novel antiseizure medications (ASMs).

Characteristics	Fenfluramine	Cannabidiol	Cenobamate
Indications	DS and LGS	Focal epileptic seizures associated with DS, LGS, and TSC [80]	Adjunctive treatment in patients with uncontrolled focal seizures
Dose	0.2, 0.4, and 0.7 mg/kg/day	2.5–60 mg/kg/day [81]	200–400 mg/day [82]
Mechanism	Increasing extracellular 5‐HT levels Activating sigma‐1 receptor [83]	The agonist and antagonist effects on ionic channels, neurotransmitter transporters, and multiple 7‐transmembrane receptors [81]	Blockade of persistent sodium currents Increasing both phasic and tonic GABA inhibition [84]
AUC	Increased dose proportionally (37316680)	Increased less than dose proportionally [85]	Increased more than dose proportionally [86]
Bioavailability (%)	75–83 [83]	6–20 [87]	88 [82]
Time to maximum concentration	3 h [83]	4–5 h [87]	0.8–4 h [88]
Metabolism	CYP1A2, CYP2B6 and CYP2D6, and, CYP2C9, CYP2C19 and CYP3A4/5 to a less extent [83]	CYP3A4, CYP2C19, and CYP2D6	UGT2B7 (minor extent UGT2B4) CYP2E1, 2A6, and 2B6, (2C19 and 3A4/5 to a lesser extent) [89]
Terminal half‐life	Approximately 20 h [83]	Approximately 60 h [87]	Range from 30 to 76 h with increasing dose [88]
Time to steady‐state concentration	4–5 days [83]	Approximately 2 days [87]	Approximately 2 weeks
Elimination	>90% in urine <5% in the feces [83]	70%–75% by liver [90]	88% in urine [91]
Affects the metabolism of other ASMs	ASMs containing stiripentol [83]	Clobazam and stiripentol [92]	Lacosamide; clobazam; phenytoin; carbamazepine [14]
>50% responder rate	DS: 38%–73% LGS: 25%–28% [83]	DS: 43% [93] LGS: 44% [94] TSC: 40% [95]	50.4% during the 6‐week maintenance phase [96]
Seizure freedom rate	DS: 2%–13% LGS: 1% [83]	DS: 5% [93]	27.5% during the 6‐week maintenance phase [96]
Predominant adverse effects	Diminished appetite, fever, fatigue, and diarrhea [83]	Somnolence, diarrhea, decreased appetite, and increased serum aminotransferases [97]	Somnolence, dizziness, fatigue, and headache [98]

Abbreviations: 5‐HT, 5‐hydroxytryptamine; ASMs, antiseizure medications; AUC, area under the concentration‐time curve; CBD, cannabidiol; CYP, cytochrome P450; DS, Dravet syndrome; GABA, γ‐aminobutyric acid; LGS, Lennox Gastaut syndrome; TSC, tuberous sclerosis complex; UGT, UDP‐glucuronosyltransferase.

Clinical evidence has well supported the efficacy of these ASMs to treat epilepsy. The relevant clinical studies have been summarized in Table 2. In a 14‐week randomized, double‐blind, placebo‐controlled trial, FFA demonstrated remarkable efficacy in treating DS, with high‐dose FFA reducing seizure frequency by 62.3% and low‐dose FFA by 32.4% [76]. FFA has been associated with lower mortality rates among individuals with DS [101]. FFA also showed significant efficacy in reducing seizures in patients with LGS [102]. The primary adverse effects of FFA include decreased appetite, fever, fatigue, and diarrhea [83].

**TABLE 2:** Summary of clinical trials of novel epilepsy therapy.

Therapy	Trials	Results	Mechanism	Refs
FFA	Follow up 13 patients with LGS for 20 weeks	There was a 53% median reduction in convulsive seizures	Acts as an agonist on 5‐HT2 and sigma‐1 receptors. It promotes serotonin release and GABA transmission while inhibiting glutamatergic activity	[103]
FFA	Follow up 119 patients with DS for 14 weeks	In the FFA 0.7 mg/kg group, seizure frequency was reduced by 74.9%, whereas in the FFA 0.2 mg/kg group, it was reduced by 42.3%		[104]
FFA	Follow up 119 patients with DS receiving stiripentol‐inclusive regimens for 12 weeks	There was a 54.0% reduction in mean monthly convulsive seizures in patients treated with FFA 0.4 mg/kg/day Adverse reactions: decreased appetite, fatigue, diarrhea, pyrexia, etc.		[105]
FFA	Follow up 52 patients with DS for 9 months	Convulsive seizures were reduced by 77.4% on average Adverse reactions: decreased appetite		[106]
FFA	Follow up 232 patients with DS for 1–2 years	Convulsive seizure frequency decreased by 66.8% compared to baseline Adverse reactions: pyrexia, nasopharyngitis, and decreased appetite		[107]
FFA	Follow up 58 patients with DS for 1 year	From pre‐randomization baseline, seizures were reduced by 75% on average FFA improved daily execution functions		[108]
FFA	Follow up 263 patients with LGS for 14 weeks	Drop seizures: 0.7 mg/kg/day FFA reduced seizures by 26.5%, whereas 0.2 mg/kg/day FFA reduced seizures by 14.2% GTCS: FFA 0.7 mg/kg/day caused a 45.7% reduction in seizure frequency, whereas FFA 0.2 mg/kg/day caused a 58.2% decrease in seizure frequency Adverse reactions: decreased appetite, somnolence, fatigue, etc.		[102]
FFA	Follow up 247 patients with DS for 12 weeks	In patients with DS, FFA can significantly reduce seizure burden on a daily basis Adverse reactions: decreased appetite, pyrexia, upper respiratory tract infection, diarrhea, and fatigue		[109]
FFA	Follow up 247 patients with LGS for 12 months	There was a median reduction of 48.8% and 35.8% in GTCS and tonic seizures with treatment, respectively Adverse reactions: decreased appetite and fatigue		[110]
FFA	Follow up 10 patients with Sunflower syndrome for 2 years	There was a median reduction in seizure frequency of 33% among patients Adverse reactions: reduced appetite and fatigue		[111]
FFA	Follow up 61 children with DS for 15 weeks	FFA treatment improved everyday executive function in children (<5 years) with DS		[112]
CBD	Follow up 15 patients with DRE	CBD can improve symptoms in epilepsy patients	Regulating neuronal membrane potential, ion channels, and neurotransmitter release	[113]
CBD	Follow up 15 patients with DRE for 4.5 weeks	87.5% of patients have improved disease status		[114]
CBD	Follow up 213 patients with DRE for 12 weeks	There was a 36.5% reduction in monthly motor seizures Adverse reactions: somnolence, decreased appetite, diarrhea, etc.		[115]
CBD	Follow up 180 patients with DS for 18 weeks	CBD reduced convulsive seizures by at least 50% in 43% of patients Adverse reactions: diarrhea, vomiting, and fatigue		[93]
CBD	Follow up 34 patients with LGS for 18 weeks	Drop seizure frequency decreased by 43.9% from baseline on average Adverse reactions: diarrhea, somnolence, pyrexia, etc.		[94]
CBD	Follow up 34 patients with DS for 12 weeks	The most common CBD‐related adverse effects were pyrexia, somnolence, decreased appetite, etc.		[116]
CBD	Follow up 225 patients with LGS for 14 weeks	Drop‐seizure frequency was reduced by 41.9% in the 20 mg CBD group and 37.2% in the 10 mg CBD group during treatment Adverse reactions: somnolence, decreased appetite, diarrhea, etc.		[117]
CBD	Follow up 139 patients with DRE for 48 weeks	CBD improved frequency and severity of seizures		[118]
CBD	Follow up 264 patients with DS for 48 weeks	Total seizures decreased by 39%–51% from baseline		[119]
CBD	Follow up 366 patients with LGS for 48 weeks	As of Week 48, the median reduction in drop seizure frequency was between 48% and 60% from baseline Adverse reactions: diarrhea, somnolence, convulsion, etc.		[120]
CBD	Follow up 53 patients with DRE for 1 year	Mood and quality of life may be improved by CBD		[121]
CBD	Follow up 607 children with DRE for 144 weeks	Adding CBD to the treatment reduced median monthly major motor seizures by 50% and total seizures by 44% at 12 weeks, with continued reductions through 96 weeks Adverse reactions: somnolence and diarrhea		[122]
CBD	Follow up 16 children with DRE for 12 weeks	In pediatric patients with DRE, CBD showed a potent seizure‐reducing effect and was safe and tolerable		[123]
CBD	Follow up 38 children with DRE for 1 year	There are no adverse effects associated with CBD that affect cognition or adaptive function		[124]
CBD	Follow up 198 patients with DS for 14 weeks	Similar clinically relevant reductions in convulsive seizure frequency were observed with CBD at doses of 10 and 20 mg/kg/day		[125]
CBD	Follow up 244 patients with TSC for 16 weeks	In comparison with placebo, cannabidiol significantly reduced seizures associated with TSC. Doses of 25 mg/kg/day had a better safety profile than 50 mg/kg/day		[95]
CBD	Follow up 368 patients with LGS for 826 days	For total seizures, the median percent reduction from baseline ranged from 48% to 68% Adverse reactions: convulsion, diarrhea, pyrexia, somnolence, etc.		[126]
CBD	Follow up 235 patients with DS for 4 weeks	A CBD treatment effect can be seen within a week of starting it		[127]
CBD	Follow up 315 patients with DS for 444 days	For total seizures, the median reduction from baseline was 49%–84% Adverse reactions: anorexia, diarrhea, pyrexia, somnolence, etc.		[128]
CBD	Follow up 48 patients with DEE for 6.5 months	Adverse reactions: dryness, pain, and somnolence at the application site		[129]
CBD	Follow up 35 children with DRE for 3 months	CBD reduced IEDs and improved sleep microstructure in children with DRE		[130]
CBD	Follow up 201 patients with TSC for 16 weeks	Reductions in seizure frequency ranged between 54% and 68% on a median basis		[131]
CBD	Follow up 188 patients with TSC for 16 weeks	After 6–10 days of treatment, the treatment began to have an effect		[132]
CBD	Follow up 188 patients with DRFE for 2 years	In 60.8% of patients, seizures were reduced by at least 50%		[132]
CBD	Follow up 44 patients with DRFE for 12 weeks	At 12 weeks, 79.5% of patients had seizures reduced by more than 50%		[133]
CNB	Follow up six patients with photosensitive epilepsy	Cenobamate suppresses IPS‐induced PPR in patients with photosensitive epilepsy	Functions as a positive allosteric modulator of GABAA receptors, consistently blocking sodium channel currents and enhancing GABAergic signaling	[134]
CNB	Follow up 437 patients with focal DRE for 12 weeks	The frequency of epileptic seizures decreased by 35.5% in the 100 mg group, 55% in the 200 mg group, and 55% in the 400 mg group Adverse reactions: somnolence, dizziness, headache, etc.		[135]
CNB	Follow up 1347 patients with uncontrolled focal seizures for 12 weeks	Adverse reactions: somnolence, dizziness, fatigue, etc.		[136]
CNB	Follow up 222 patients with uncontrolled focal seizures for 12 weeks	50.4% of subjects in the CNB group experienced a reduction of ≥50% in seizure frequency Adverse reactions: somnolence, dizziness, headache, nausea, and fatigue		[96]
CNB	Follow up 147 patients with uncontrolled focal seizures for 12 weeks	Adults with treatment‐resistant focal seizures taking 1–3 ASMs were found to tolerate adjunctive cenobamate treatment for 7.8 years		[137]
CNB	Follow up 49 patients with uncontrolled focal seizures for 8 years	In 45% of patients, seizures were reduced by 75%, in 29% by 90%, and in 16%, seizures were completely eliminated		[138]
CNB	The data were pooled from 1844 participants	In adults with focal seizures, long‐term individual retention with cenobamate has been observed		[139]
CNB	Follow up patients with uncontrolled focal seizures for 4 weeks	Titration reduced seizure frequency with initial efficacy observed before reaching the target dose		[140]
CNB	Follow up 355 patients with focal DRE for 48 months	Over the course of 36–48 months, 16.4% of observed patients achieved 100% seizure reduction and 39.1% achieved 90% seizure reduction Adverse reactions: dizziness, somnolence, fatigue, headache, etc.		[141]
CNB	Follow up 394 patients with uncontrolled focal seizures for 18 weeks	In the cenobamate‐treated structural cause and unknown cause groups, seizure frequency was significantly reduced		[142]
CNB	Follow up 442 patients with uncontrolled focal seizures for 7 years	With adjunctive cenobamate treatment, patients with uncontrolled focal seizures exhibit a low rate of cognitive or psychiatric adverse reactions		[143]
LITT	Follow up 145 patients with MTLE for 2 years	2‐year seizure outcomes 58.4% and 57.2% for Engel I and International League Against Epilepsy 1/2, respectively. Adverse events were seen in 16.5% patients	Uses MRI‐guided laser light to induce precise thermal ablation (coagulative necrosis) of the EZ	[144]
LITT	Follow up 244 patients with drug‐resistant MTLE	There were no significant differences in memory preservation or seizure control between the MRgLITT group and the open surgery group		[145]
LITT	Follow up 135 patients with drug‐resistant MTLE for 3.5 years	LITT is not effective in reducing SUDEP incidence in patients with DRE		[146]
LITT	Follow up 39 patients with PVNH	MRgLITT improves the seizure control outcomes of PVNH‐associated epilepsy		[147]
LITT	Follow up 268 patients with drug‐resistant MTLE for 2 years	Engel I or II outcomes were achieved in 74.2% at 1 year, 75.0% at 2 years, and 66.0% at the last follow‐up		[148]
LITT	Follow up 103 patients with epilepsy for ≥1 year	LITT is a less‐invasive surgical alternative for corpus callosotomy, associated with similar seizure outcomes, lower blood loss, shorter hospital stays, and lower complication rates, when compared with the open craniotomy approach		[149]
LITT	Follow up 370 children with DRE for 1 year	Seizure outcome of MRgLITT at 1 year posttreatment was inferior to open surgery		[150]
LITT	Follow up 27 patients with MTLE for 1.9 years at average	Seizure outcome after LITT in patients with MTLE was associated significantly with the extent of cluster ablation in the amygdalohippocampal complex		[151]
LITT	Follow up 48 patients with MTLE for 2 years	60.4% patients achieved an Engel Class I outcome, whereas 22.9% had one to three seizures/year		[152]
LITT	Follow up 61 patients with BOSD‐related epilepsy	The seizure‐free rates were comparable between the MRgLITT group and open surgery group		[153]
LITT	Follow up 14 patients with insular epilepsy for 6 months	64.3% patients achieved complete seizure freedom, 14.3% achieved seizure freedom but retained auras, and 21.4% saw no improvement in their epilepsy		[154]
LITT	Follow up 36 patients with DRE for 6 months	The study is performed to compare the effectiveness and safety of CC to CCA. This early experience suggests CCA has similar outcomes to traditional CC		[155]
LITT	Follow up 47 patients with HH	The overall Engel Class I rate is 68.1%		[156]
LITT	Follow up 10 patients with DRE	71% patients experienced freedom from drop attacks at longest follow‐up and 57% of cases showed improvement in other seizure semiologies		[157]
LITT	Follow up 27 patients with ETLE	MRgLITT for ETLE allows for ASMs reduction in a significant portion of patients and complete ASMs withdrawal in a subset of them		[158]
LITT	Follow up five children with DRE	The study has demonstrated the feasibility of a minimally invasive approach for completion hemispherotomy using MRgLITT		[159]
RFTC	Follow up 38 patients with MTLE	SEEG‐guided RFTC preserved cognitive and visual function better in dominant‐side MTLE than in ATL, with comparable seizure control and quality of life outcomes	Involves stereotactic implantation of electrodes for recording and then delivering radiofrequency current through the same electrodes to create targeted thermal lesions	[160]
RFTC	Follow up 39 patients with MTLE	After RFTC within the dominant MTLE, 20% of patients experienced a decline in verbal memory and 10% in visual memory. 7% declined in verbal memory and 10% in visual memory post‐RFTC outside the dominant MTLE		[161]
RFTC	Follow up 41 patients with DRE	48.7% patients experienced a seizure frequency decrease of at least 50%, which was over 80% in eight of them		[162]
RFTC	Follow up five patients with DRE	Postprocedural sustained seizure freedom was detected in four cases		[163]
RFTC	Follow up 43 patients with HH with a median of 38 months	12 months after their last SRT, 60% of patients were seizure‐free		[164]
RFTC	Follow up four patients with PVNH	Two patients experienced significant seizure reduction and improvement in psychiatric symptoms, one showed partial improvement, and one had no significant benefit		[165]
RFTC	Follow up 131 patients with HH for 3 years	Seizure freedom was obtained in 116 patients (88.6%) for gelastic seizures		[166]
RFTC	Follow up 19 patients with refractory insular epilepsy for 1–12 years	Seizure‐free outcome was achieved in 10 patients (53%)		[167]
RFTC	Follow up 88 patients with HH for 3.3 years at average	85.2% of patients achieved gelastic seizure remission		[168]
RFTC	Follow up 23 patients with DRE for 2–119 months	8 patients experienced a ≥50% decrease of seizure frequency after RFTC		[169]
RFTC	Follow up 162 patients with DRE for 1 year	25% of patients were seizure‐free at 2 months and 7% at 1 year. Overall, 67% of responders at 2 months and 48% at 1 year		[170]
RFTC	Follow up 20 patients with HH for 19 months at average	15% of the patients became seizure free, 40% experienced a ≥80% reduction of their seizure frequency		[171]
RFTC	Follow up 69 patients with HH for 41 months at average	Seizure freedom was obtained by 69.57% patients for clinical seizures		[100]
RFTC	Follow up nine children with DRE	88.9% patients achieved complete remission after the final operation at half‐year follow‐up		[172]
RFTC	Follow up 70 patients with TLE for 1 year	SEEG‐guided RF‐TC is not as effective as ATL in TLE		[173]
RFTC	Follow up 22 patients with MTLE for 12–48 months	Sixteen patients (72.72%) were seizure‐free		[174]
RFTC	Follow up 121 patients with refractory focal epilepsy for 18.3 months at average	67.8% of patients were responders and 44.6% were seizure free		[175]
RFTC	Follow up 25 patients with HH	Complete seizure freedom was achieved in 19 patients (76.0%)		[176]
RFTC	Follow up 48 patients with refractory focal epilepsy	Discernible cognitive impairment following RFTC was not evidenced		[177]
RFTC	Follow up 31 patients with refractory focal epilepsy	Seizure outcome at the last follow‐up visit (mean 18 months) showed seizure freedom in two patients (6.5%) and ≥50% reduced seizure frequency in 20 patients (64.5%)		[178]
RFTC	Follow up 55 patients with refractory focal epilepsy for 30.9 months at average	14 patients (45.2%) showed at least a 50% reduction in seizure frequency (responders), and 8 were seizure free (25.8%)		[179]
RFTC	Follow up nine patients with HH for 6 months	The seizure frequency within 6 months decreased postoperatively with a mean reduction in seizures of 78%		[180]
RFTC	Follow up 16 patients with giant HH for 23 months at average	13 patients (81.3%) achieved freedom from gelastic seizures		[181]
RFTC	Follow up 100 patients with HH for 23 months at average	86% of patients achieved freedom from gelastic seizures		[182]
RFTC	Follow up 89 patients with refractory focal epilepsy	Sustained seizure freedom occurred after RFTC in 16 patients (18.0%)		[183]
RFTC	Follow up 28 patients with MTLE for 5 years	The proportions of patients categorized as Engel I between 1 and 5 years after surgery were 72.41% (12 months), 67.86% (18 months), 62.07% (24 months), 50.00% (36 months), 42.86% (48 months), and 42.86% (60 months), respectively		[184]
RFTC	Follow up 150 patients with HH	122 patients (81.3%) achieved freedom from gelastic seizures		[185]
RFTC	Follow up 24 patients with PVNH	17 patients (71%) responded (ILAE class 1–4) after SEEG‐guided RFTC		[186]
VNS	Follow up five patients with CPS for 2 weeks	VNS reduced seizure frequency, duration, or intensity Adverse reactions: a tingling sensation in the throat and hoarseness	Modulates neural activity by electrically stimulating the left vagus nerve in the neck. It acts through afferent pathways to influence brainstem, thalamic, and cortical regions, producing acute changes in neural activity and chronic changes in circuit function and neurochemistry	[187]
VNS	Follow up 14 patients with refractory partial seizures for 35 months	There was a 50% or greater reduction in seizure frequency in 35.7% of patients Two patients have been seizure‐free for over 1 year Adverse reactions: hoarseness and a tingling sensation		[188]
VNS	Nine CPS patients	High stimulation led to an additional 14.3% reduction, whereas low stimulation led to a 25.4% reduction in seizure frequency		[189]
VNS	Follow up 67 patients with refractory partial seizures for 14 weeks	Among patients receiving high VNS, 38.7% had seizure frequency reduced by more than 50% Among patients receiving low VNS, 19.4% had seizure frequency reduced by more than 50% Adverse reactions: hoarseness, coughing, and throat pain		[190, 191]
VNS	Follow up 114 patients DRE for 14 weeks	In the high stimulation group, seizure frequency was reduced by 24.5% vs. 6.1% in the low stimulation group		[192]
VNS	Follow up 196 CPS or secondarily generalized seizure patients for 3 months	In the high stimulation group, total seizure frequency was reduced by 28% on average In the low stimulation group, total seizure frequency was reduced by 15% on average		[193]
VNS	Follow up 24 resistant generalized epilepsy patients for 3 months	About half of the participants experienced a 50% reduction in seizure frequency Adverse reactions: coughing		[194]
VNS	Follow up 15 children with epileptic encephalopathies for 1 year	Seizure frequency decreased by 17% on average Verbal performance and general behavior were improved		[195]
VNS	Follow up 199 patients with DRE for 15 months	There was a greater than 50% reduction in seizures among 39 subjects. Overall, 21% of subjects had a greater than 75% reduction in seizures, and 2% remain seizure‐free		[196]
VNS	Follow up 45 DRE patients for 1 year	21 of 31 participants in the study had a seizure decrease of more than 50% at 1 year Adverse reactions: hoarseness, voice alterations, coughing, and so on		[197]
VNS	Follow up 195 DRE patients for 12 months	Among 35% of subjects, seizures were reduced by at least 50%		[198]
VNS	Follow up 13 patients with LGS for 6 months	In three patients, seizures were reduced by more than 90%, two by more than 75%, and one by more than 50%		[199]
VNS	Follow up 50 LGS patients for 6 months	Across the 3 months, total seizures were reduced by 42%, 58.2%, and 57.9% Adverse reactions: voice alteration and coughing		[200]
VNS	Follow up 95 patients with DRE	45% of patients experience a reduction in seizures of at least 50%		[201]
VNS	Follow up 13 children with DRE for 1 year	Six of eight patients experience a reduction in seizures of at least 50%		[202]
VNS	Follow up six patients with DRE for 48 months	The seizure frequency reduction is 60%		[203]
VNS	Follow up 13 patients with DRE for 22 months	38.4% of patients had a 50% or more reduction in seizure frequency		[204]
VNS	Follow up 269 patients with DRE for 21 months	The median seizure frequency reduction is 43.3%		[205]
VNS	Follow up 16 patients with DRE for 12 months	The median seizure frequency reduction is 58%		[206]
VNS	Follow up 131 patients with DRE for 33 months	There was a 50% reduction in seizure frequency in 50% of patients Adverse reactions: hoarseness and gagging		[207]
VNS	Follow up 40 DRE children for 2 years	Eleven subjects experienced a reduction in seizures of at least 50%		[208]
VNS	Follow up 15 DRE children for 9 months	Among 15 children, 6 showed a 50% or greater reduction in seizure frequency; 1 became seizure‐free VNS improves seizure intensity, quality of life, emotions, etc.		[209]
VNS	Follow up 15 DRE children for 9 months	Slow wave sleep is increased by VNS, which counteracts epilepsy's adverse effects on sleep		[210]
VNS	Follow up 20 patients with DRE	VNS decreased IEDs in 80% of patients		[211]
VNS	Follow up seven patients with Rett syndrome for 12 months	Six patients had a 50% or more reduction in seizure frequency Improvement of alertness		[212]
VNS	Follow up 19 patients with DRE for 6 years	There was a 50% reduction in seizure frequency adverse reactions: hoarseness and coughing		[213]
VNS	Follow up 31 patients with refractory partial and generalized seizures for 4 years	Overall, 53.3% of patients had a 50% or more reduction in seizure frequency Improvement of alertness and feeling Adverse reactions: hoarseness, cough, and vomiting		[214]
VNS	Follow up 26 children with DRE for1.5–8.5 years	Overall, 54% of patients had a 50% or more reduction in seizure frequency		[215]
VNS	Follow up eight patients with DRE for 36 months	Overall, 75% of patients had a 50% or more reduction in seizure frequency Decreased IEDs		[216]
VNS	Follow up 28 children with epileptic syndrome for 24 months	Overall, 68% of patients had a 50% or more reduction in seizure frequency Improvement of alertness, playfulness, interaction, and sleep time Adverse reactions: throat discomfort and hoarseness		[217]
VNS	Follow up 30 LGS patients for 17–123 months	The median seizure frequency reduction is 60.6% Shorter ictal or postictal and improved alertness Adverse reactions: drooling and voice alteration		[218]
VNS	Follow up 19 DRE and TSC patients for 8.5 months–9.6 years	Overall, 72% reduction in average seizure frequency		[219]
VNS	Follow up 26 patients with nonfocal epilepsy or LGS for 2 years	In the first year, the average seizure reduction was 23%, and in the second year, it was 22%		[220]
VNS	Follow up 41 children with DRE for 20 weeks	Among participants with VNS, 26% experienced a 50% or greater reduction in seizure frequency Seizure severity was improved		[221]
VNS	Follow up 41 DRE patients for 5 months	26% patients ≥50% Improvement of mood, especially depression		[222]
VNS	Follow up 112 DRE patients for 12 months	24% patients ≥50% Improving long‐term health‐related quality of life Transient vocal cord paralysis		[223]
VNS	Follow up 74 children with DRE for 1 year	The frequency of seizures was reduced by 50% for 74.3% of patients, and postictal and ictal activity were also improved		[224]
ANT‐DBS	Follow up 100 patients with DRE for 2 years	The median seizure frequency of patients had reduced by 56% within 2 years; 54% of patients had reduced seizure frequency by at least 50%, and 14 patients were seizure‐free for at least 6 months	Delivers electrical pulses to the ANT, a key node in the circuit of Papez, to modulate abnormal neural activity and disrupt seizure networks	[225]
ANT‐DBS	Follow up nine patients with DRE for 12 months	Seizure reduction rate was 57.9% on average Verbal fluency tasks and delayed verbal memory were significantly improved		[226]
ANT‐DBS	Follow up 15 patients with DRE for 27 months	The mean reduction of seizure frequency is 70.4%		[227]
ANT‐DBS	Follow up 105 patients with DRE for 5 years	At 5 years, the median seizure reduction was 69%		[228]
ANT‐DBS	Follow up 18 patients with DRE for 12 months	There was a 50% reduction in total seizure frequency in four patients and a 50% reduction in focal seizures in five patients		[229]
ANT‐DBS	Follow up 110 patients with DRE for 10 years	Seven years later, the median seizure frequency had decreased by 75% from baseline, and the most severe type of seizure, focal to bilateral tonic–clonic, had decreased by 71%		[230]
ANT‐DBS	Follow up 191 patients with DRE for 2 years	There was a median reduction of 33.1% in seizure frequency Adverse reactions: changes in seizure, memory impairment, depressive mood, etc.		[231]
RNS	Follow up 119 patients with refractory partial seizures for 96 weeks	The average frequency of epileptic seizures in the treatment group decreased by 37.9% Overall quality of life improved significantly	Uses a closed‐loop system to detect and respond with stimulation to epileptic activity from electrodes placed near the seizure focus	[232]
RNS	Follow up 191 patients with refractory partial seizures for 2 years	After 1 year and after 2 years, 44% and 53% of seizures were reduced, respectively		[233]
RNS	Follow up 230 patients with refractory partial seizures for 7 years	According to the long‐term study, the median percent reduction in seizure frequency was 44% at 1 year, 53% at 2 years, and 48%–66% at 3–6 years postimplant Improvements in QOL Adverse reactions: infection		[234]
RNS	Follow up 191 patients with medically resistant focal epilepsy for 2 years	QOL improved		[235]
RNS	Follow up 111 patients with MTLE for 6 years	There was a median reduction of 70% in seizure frequency. Overall, 29% of the subjects experienced a seizure‐free period of at least 6 months, and 15% experienced a seizure‐free period of at least 1 year Adverse reactions: infection		[236]
RNS	Follow up 126 patients with seizures of neocortical onset for 6 years	Among patients with frontal and parietal seizure onsets, the median percent seizure reduction was 70%, for those with temporal neocortical onsets, it was 58%, and for those with multilobar onsets, it was 51%		[237]
FUS	Follow up two patients with uncontrolled focal seizures for 2 years	Both patients experienced a significant reduction in seizure frequency	Concentrates ultrasound energy to achieve thermal ablation of EZ, providing a noninvasive alternative to surgical resection	[238]
FUS	Follow up six patients with DRE for 9 months	There was a 50% reduction in seizure frequency at average posttreatment; improvement lasted from weeks to several months		[239]
FUS	Follow up patients with DRE	Investigate patient tolerability and efficacy of pulsed LIFUS in patients with drug‐resistant TLE No results		NCT03868293
FUS	Follow up patients with DRE	Evaluating the safety and efficacy of MRgFUS in patients with DRE No results		NCT02804230
FUS	Follow up 16 patients with DRE for 24 weeks	Evaluating the safety and efficacy of LIFUS in patients with DRE No results		NCT06492720
FUS	Follow up 10 patients with DRE for 12 months	Evaluating the safety and efficacy of MRgFUS in patients with DRE No results		NCT03417297
FUS	Follow up eight patients with DRE for 12 weeks	Evaluating the safety and efficacy of LIFUS in patients with DRE No results		NCT06388707
FUS	Follow up patients with DRE	Evaluating the safety and efficacy of FUS in patients with DRE No results		NCT06292494
FUS	Follow up 10 patients with DRE for 12 months	Evaluating the safety and efficacy of MRgFUS in patients with DRE No results		NCT05032105
FUS	Follow up patients with TLE	Evaluating the safety and efficacy of LIFUS in patients with TLE No results		NCT03657056
FUS	Follow up patients with TLE	Evaluating the safety and efficacy of LIFUS in patients with TLE No results		NCT02151175
FUS	Follow up patients with DRE	Evaluating the safety and efficacy of FUS in patients with DRE No results		NCT05947656
FUS	Follow up patients with DRE	Evaluating the safety and efficacy of FUS in patients with DRE No results		NCT04999046
Cell therapy	Follow up 60 patients with DRE	Evaluating the safety and efficacy of autologous MSC application for the therapy of DRE No results	Involves the transplantation of inhibitory neuron progenitors into the EZ. These cells migrate, integrate into host circuits, mature into functional GABAergic interneurons, and enhance synaptic inhibition	NCT02497443
Cell therapy	Follow up subjects with drug‐resistant bilateral mTLE	Evaluating the efficacy and safety of inhibitory nerve cell therapy in DRE patients No results		NCT06422923
Cell therapy	Follow up subjects with drug‐resistant unilateral mTLE	Evaluating the efficacy and safety of inhibitory nerve cell therapy in DRE patients No results		NCT05135091
Gene therapy	Follow up patients with DRE	Evaluating the safety of in vivo lentiviral engineered K+ channel gene therapy for DRE No results	Uses viral vectors to introduce a functional copy of a gene to compensate for a loss‐of‐function mutation	NCT04601974
Gene therapy	Follow up subjects with mTLE	Evaluating the efficacy and safety of AAV9‐hSyn1‐miGRIK2 therapy in DRE patients No results		NCT06063850
Gene therapy	Follow up children with DS	Evaluating the efficacy and safety of an AAV9‐delivered therapy in children With SCN1A‐positive DS No results		NCT06112275
Gene therapy	Follow up children with DS	Evaluating the efficacy and safety of an AAV9‐delivered therapy in children With SCN1A‐positive DS No results		NCT06283212
Gene therapy	Follow up children with DS	Evaluating the efficacy and safety of an AAV9‐delivered therapy in children With SCN1A‐positive DS No results		NCT05419492

Abbreviations: ANT‐DBS, deep brain stimulation of the anterior nucleus of the thalamus; ATL, anterior temporal lobectomy; BOSD, bottom‐of‐sulcus dysplasia; CBD, cannabidiol; CC, corpus callosum; CCA, corpus callosum ablation; CNB, cenobamate; CPS, complex partial seizures; DEE, developmental and epileptic encephalopathy; DRE, drug‐resistant epilepsy; DRE, drug‐resistant epilepsy; DRFE, drug‐resistant focal epilepsy; DS, Dravet syndrome; ETLE, extra‐temporal lobe epilepsy; EZ, epileptogenic zone; FFA, fenfluramine; FUS, focused ultrasound; GABA, gamma‐aminobutyric acid; GTCS, generalized tonic–clonic seizure; HH, hypothalamic hamartoma; IEDs, interictal epileptiform discharges; LGS, Lennox Gastaut syndrome; LITT, laser interstitial thermal therapy; MRgFUS, magnetic resonance‐guided focused ultrasound; MRI, magnetic resonance imaging; MTLE, mesial temporal lobe epilepsy; NH, nodular heterotopy; PVNH, periventricular nodular heterotopia; QOL, quality of life; RNS, responsive neurostimulation; SEEG‐guided RFTC, stereoelectroencephalography‐guided radiofrequency thermocoagulation; TLE, temporal lobe epilepsy; TSC, tuberous sclerosis complex; VNS, vagus nerve stimulation.

Adjunctive CBD demonstrates significant efficacy in treating seizures associated with specific DEE. CBD significantly reduced seizure frequency in patients with DS. At doses of 10 and 20 mg/kg/day, the reductions were 48.7% and 45.7%, respectively. This was much greater than the 26.9% reduction observed with placebo [125]. Long‐term CBD treatment led to sustained median reductions in drop seizure frequency of 48%–71% over 156 weeks in patients with LGS [126]. In patients with TSC, CBD significantly reduced seizure frequency. The reductions were 48.6% at 25 mg/kg/day and 47.5% at 50 mg/kg/day, compared to a 26.5% reduction with placebo [95]. The ≥50% responder rates (≥50% seizure reduction) were consistently higher with CBD across all syndromes. Common adverse effects of CBD include somnolence, diarrhea, reduced appetite, and elevated serum aminotransferases [97].

In two Phase 2 trials, CNB significantly reduced seizure frequency in adults with uncontrolled partial‐onset seizures [96, 98]. Patients receiving CNB in the 200 and 400 mg dose groups experienced a median seizure reduction of over 50%. During the 12‐week maintenance phase, the seizure‐free rate ranged from 11% to 21% [98]. The primary adverse effects of CNB include somnolence, dizziness, and fatigue [98]. CNB undergoes hepatic metabolism and is predominantly excreted renally, necessitating caution in patients with hepatic or renal impairment. As a liver enzyme inhibitor, CNB may reduce the clearance of certain ASMs, necessitating careful monitoring for potential drug interactions and adverse effects when co‐administered with other ASMs [14].

#### 4.1.2 New Developments in Traditional ASMs

Beyond novel compounds, optimizing existing ASMs is critical. Emergency treatment options for cluster seizures were historically limited to rectal diazepam administration or oral benzodiazepines, whereas intravenous diazepam was reserved for SE. These approaches presented practical challenges in everyday scenarios, often leading to delayed or suboptimal treatment. Recently, the approval of benzodiazepine nasal sprays for cluster seizure rescue has provided a promising alternative. Intranasal formulations, such as diazepam nasal spray (Valtoco) and midazolam nasal spray (Nayzilam), are designed for rapid absorption, avoiding first‐pass metabolism [240]. Nasal formulations are characterized by rapid onset, ease of use, and patient acceptability, underscoring their unique value in emergency situations [99] (Figure 2D).

Diazepam nasal spray typically takes effect within 2–10 min, with seizures halting in a median of 4 min and 75% resolving by 11 min [241]. Furthermore, these nasal preparations offer good stability and safety, with minimal variability in pharmacokinetics across individuals, making them suitable for both ictal and interictal use [242]. No severe treatment‐emergent adverse events were found to be related to the therapy [99]. Notably, nasal sprays also contribute to higher 24‐h seizure‐free rates [243].

Buccal midazolam is an oromucosal solution in prefilled syringes that was approved by the European Medicines Agency in 2011 [240]. Both intranasal and buccal benzodiazepines are effective alternatives to rectal formulations for acute seizure management. An indirect comparison meta‐analysis indicates that both intranasal and buccal midazolam are effective in terminating acute seizures, with no significant difference in efficacy [244]. In terms of safety and tolerability, the same analysis revealed no significant difference in serious adverse events between the two routes [244]. However, intranasal midazolam may lead to a higher incidence of nasal discomfort, which can be attributed to formulation characteristics such as low pH and the presence of organic solvents [6]. In contrast, buccal administration may be compromised by factors like hypersalivation, jaw clenching, or swallowing difficulties during a seizure, potentially affecting drug absorption [6]. Additionally, ready‐to‐use nasal delivery devices offer simpler administration compared to buccal solutions that often require measurement from ampoules [245]. Despite these observations, no randomized controlled trials have directly compared intranasal benzodiazepines with buccal midazolam for this indication. Therefore, well‐designed head‐to‐head trials are urgently needed to provide direct comparative evidence.

However, the efficacy of drug therapy remains constrained by the diverse etiologies, genetic variability, and pathogenic mechanisms of epilepsy. Although novel ASMs can reduce seizure frequency, they do not offer a cure for the disease. Adverse effects and potential drug interactions further limit the benefits for certain patients. Moreover, complex dosing regimens and the high pill burden often hinder adherence, contributing to breakthrough seizures in approximately 45% of cases [246]. Consequently, alternative and complementary approaches (e.g., surgical interventions) are critical for eligible patients with refractory epilepsy.

### 4.2 Advances in Epilepsy Surgery

For patients with DRE, surgical intervention represents a potentially curative approach. Resective open surgery has been the standard treatment for focal epilepsies with identifiable lesions, achieving high rates of seizure freedom [247]. However, such procedures are associated with significant morbidity. Approximately 35% of patients undergoing extensive resections experience functional deficits [248]. In recent years, considerable effort has been focused on identifying optimal interventions that maximize seizure freedom while minimizing procedural risks. Advances in imaging and electrophysiology have enhanced the precision of epileptogenic focus localization, improving surgical outcomes [249]. The introduction of minimally invasive techniques has further reduced surgical morbidity without compromising efficacy. Moreover, the integration of robotic‐assisted systems has increased the precision and efficiency of epilepsy surgery [250].

#### 4.2.1 Advances in Preoperative Evaluation for DRE

Recent advances in preoperative evaluation for DRE have significantly improved treatment outcomes, largely driven by progress in electrophysiological and neuroimaging technologies. These tools enable more precise localization of the EZ and enhance surgical efficacy [251]. This section summarizes key developments in electrophysiology and neuroimaging.

Emerging electroencephalogram (EEG) analysis techniques and biomarkers show increasing potential for clinical translation, offering new insights into epileptic focus localization and network dynamics. Among these, electrical source imaging (ESI) and high‐frequency oscillations (HFOs) have attracted considerable interest [252]. ESI using high‐density EEG has become a valuable noninvasive method in the presurgical evaluation of drug‐resistant focal epilepsy. By combining temporal and spatial EEG data with MRI‐derived head models, ESI localizes interictal and ictal activity with high specificity (88%) and sensitivity (84%), outperforming MRI or PET alone [249]. Automated spike detection combined with source localization further allows reliable EZ identification, achieving sublobar concordance rates over 80% [253]. HFOs, particularly fast ripples (250–600 Hz), are increasingly recognized as biomarkers of EZ. Meta‐analyses confirm that complete resection of HFO‐generating regions is associated with postoperative seizure freedom [254]. HFOs can now be measured noninvasively using scalp EEG and magnetoencephalography, broadening their clinical applicability [255].

Structural and functional neuroimaging are essential in the presurgical evaluation of patients with DRE. Structural MRI is routinely used to identify epileptogenic lesions [256]. Although 1.5 T and 3 T MRI remain standard in clinical practice, the introduction of 7 T MRI has substantially improved lesion detection [257]. The higher spatial resolution and contrast of 7 T MRI enable visualization of subtle abnormalities not detectable at lower field strengths [258]. For example, sequences such as T2‐weighted and susceptibility‐weighted imaging at 7 T improve delineation of mesial temporal structures and cortical malformations, increasing diagnostic yield by 31% compared to 3 T MRI [259]. Functional imaging is particularly valuable in nonlesional cases and multifocal epilepsy [260]. Recent progress in functional imaging has significantly improved localization of SZ in refractory epilepsy. Blood‐oxygen‐level‐dependent (BOLD) functional MRI (fMRI) is widely used for noninvasive mapping of eloquent cortical areas, reducing reliance on invasive procedures such as the Wada test [261]. Magnetoencephalography source imaging provides millisecond‐level temporal resolution for localizing interictal spikes [262]. Single‐photon emission computed tomography (SPECT) and PET serve as complementary functional modalities. The advantage of SPECT is its ability to capture ictal imaging, where brain areas involved in seizures show increased perfusion [263]. When ictal‐interictal subtraction images are co‐registered with MRI, SPECT achieves over 90% sensitivity in localizing the EZ, especially in TLE [264]. However, interictal SPECT offers limited localizing value [263]. In contrast, interictal fluorine‐18 FDG‐PET identifies hypometabolic regions that often correspond to the EZ [260]. Clinical studies report that FDG‐PET provides localizing information in 60%–90% of patients with TLE [265].

In conclusion, the integration of advanced technologies promises to improve postoperative seizure outcomes and increase the number of candidates who can be offered potentially curative surgery. Future progress is expected to focus on three dimensions: multimodal fusion, technological innovation, and artificial intelligence (AI) empowerment. Multimodal fusion involves combining multiple electrophysiological and neuroimaging techniques to overcome limitations inherent in any single modality [261]. For instance, simultaneous EEG‐fMRI links hemodynamic responses with electrical activity. Targeting brain areas with the strongest BOLD response during interictal discharges may improve surgical outcomes [261]. Technological innovation enhanced detection methods and devices. The integration of nanomaterials and biomedicine has enabled novel diagnostic tools for epilepsy, such as neural electrodes, contrast agents, and biosensors. They offer superior sensitivity and specificity compared to conventional approaches [266]. AI and machine learning can extract subtle patterns from large‐scale electrophysiological and imaging datasets, increasing the accuracy and efficiency of EZ localization. Studies indicate that AI‐enhanced multimodal strategies may improve surgical success rates [267]. With continued advances and integration of these technologies, epilepsy surgery is poised to achieve more precise localization of EZ, ultimately enhancing patients’ postoperative quality of life.

#### 4.2.2 Laser Interstitial Thermal Therapy (LITT)

LITT is a minimally invasive technique that uses nonionizing radiation from laser light to induce thermal damage and cell death [268]. A major drawback of early laser‐based therapeutic systems was the lack of capability to monitor heat distribution in deep‐seated lesions. This limitation was addressed by integrating MRI, allowing real‐time thermal monitoring without disrupting laser operation (Figure 2E). The approach is known as magnetic resonance‐guided LITT [269]. Since 2012, LITT has been increasingly adopted as an alternative to conventional resection in DRE, particularly for mesial temporal lobe epilepsy (MTLE) and mesial temporal lobe sclerosis [145].

Clinical evidence supports the efficacy of LITT in clinical practice. According to a multicenter study of 268 patients from 11 institutions, seizure freedom rates were 55.8% at 1 year and 52.5% at 2 years following LITT [148]. Additional studies have reported that more than 50% of patients with DRE remain seizure‐free for 2 years following LITT [144]. The most frequent adverse event is visual field deficit, often presenting as superior quadrantanopia, which likely results from injury to nearby optic radiations. The relevant clinical trials are summarized in Table 2.

Although LITT demonstrates slightly lower seizure freedom rates compared to open surgery, it offers unique advantages, including minimal invasiveness, shorter hospital stays, and reduced complication rates [144]. The procedure requires only an incision of less than 1 cm. Clinical evidence indicates that many patients with DRE prefer LITT to open surgery [270]. The median hospital stay is 1 day for LITT compared with 3–4 days for open procedures [270]. Hospitalization costs are also significantly lower with LITT ($108,332) than with open surgery ($124,012). Additionally, LITT is associated with reduced postoperative analgesic requirements [271]. Studies have further confirmed that LITT leads to less postoperative complications. A systematic review and meta‐analysis in TLE reported an overall complication rate of 6.5% for LITT compared to 11.4% for open surgery [272]. LITT also preserves the lateral temporal neocortex, which may contribute to better neurocognitive outcomes [152]. These features make LITT a good choice for patients, as it combines effectiveness, minimal invasiveness, and the preservation of future treatment options.

However, several limitations exist in the current evidence, such as small sample sizes, heterogeneous patient selection, and variability in perioperative management [148]. These factors complicate direct attribution of outcomes to LITT alone. Future studies should adopt larger, prospective, and standardized designs to better evaluate the long‐term efficacy and safety of this technique [268]. Additionally, it remains necessary to optimize surgical approaches and energy settings to minimize complication risks [273].

#### 4.2.3 Radiofrequency Thermocoagulation

Stereoelectroencephalography‐guided radiofrequency thermocoagulation (SEEG‐guided RFTC) is a minimally invasive technique for treating drug‐resistant focal epilepsy. The procedure involves stereotactic implantation of intracerebral electrodes to record electrophysiological signals and locate the EZ. Radiofrequency current is then delivered through the same electrodes to create thermal lesions and ablate the target tissue [274] (Figure 2F). SEEG‐guided RFTC has been used in various forms of DRE, such as MTLE, epilepsy related to hypothalamic hamartoma (HH), focal cortical dysplasia, periventricular nodular heterotopia (PNH), and other cortical developmental malformations [100, 166, 175].

Clinical studies support the efficacy of SEEG‐guided RFTC. Meta‐analyses and large cohort studies report 1‐year seizure freedom rates of 23%–37% and responder rates of 58%–70%, with sustained benefits observed in some patients over the long term [274, 275]. In specific etiologies such as PNH, seizure freedom rates can exceed 38% [274]. As PNH is often not amenable to open resection, SEEG‐guided RFTC may represent the most effective treatment option for related DRE. Treatment outcomes are influenced by several factors, including the presence of an MRI‐visible lesion, complete ablation of the EZ, and the number of thermocoagulation sites. Importantly, the procedure can also serve as a bridge to resection, where a positive response helps predict favorable surgical outcomes [160, 274, 275]. Reported complications include neurological deficits (e.g., motor weakness or memory impairment) in approximately 2.5% of cases, hemorrhagic events, and infections or edema [274]. The relevant clinical trials are summarized in Table 2.

The advantages of SEEG‐guided RFTC include minimal invasiveness, lower risk of major complications, and better preservation of cognitive and neurological function. It is particularly suitable for EZ located in eloquent or deep‐seated brain regions [100, 175]. In contrast to anterior temporal lobectomy (ATL), RFTC leads to fewer visual field deficits and memory impairments [173, 177]. Moreover, the same electrodes are used for both recording and ablation. This avoids the need for a second surgery and reduces both cost and procedural burden [161, 173, 177].

However, SEEG‐guided RFTC has several limitations. Its main disadvantage is a lower seizure freedom rate compared to open surgery. ATL achieves seizure freedom in 60%–80% of patients [276]. The ablation volume is limited. The absence of real‐time temperature monitoring leads to variable outcomes and possible undertreatment [277]. Future improvements aim to increase ablation precision and volume. Investigated strategies include optimizing electrode trajectories, using three‐dimensional RFTC to create larger lesions, and incorporating real‐time MRI thermometry [174]. Combining RFTC with other treatments such as LITT or responsive neurostimulation (RNS) may also enhance outcomes [100, 174].

#### 4.2.4 Endoscopic Surgery (ES)

ES employs rigid or flexible endoscopes that provide high‐definition visualization and contain integrated working channels. This system allows precise identification and removal of pathological brain tissue through natural openings or small cranial windows [278] (Figure 2G). ES has been used in the treatment of DRE, offering minimally invasive access to deep‐seated EZ [279, 280]. It is especially useful for MTLE, HH‐related epilepsy, and corpus callosotomy [278, 279, 281]. For example, the endoscopic anterior transmaxillary (eATM) approach enables targeted removal of the temporal pole and mesial structures in MTLE. Similarly, endoscopic transventricular techniques allow disconnection or resection of HH [279, 282].

Clinical studies support the efficacy of ES for DRE. In a series of four patients with MTLE treated with eATM approach, all achieved Engel Class Ia seizure freedom at 12–19 months of follow‐up, without significant neuropsychological decline [279]. A systematic review and meta‐analysis by Rizzi et al. evaluated ES in HH‐related epilepsy. The data from 88 patients showed that 47.7% achieved seizure freedom at follow‐up ≥12 months [281]. ES also demonstrated a favorable safety profile with minimal hypothalamic injury, making it a viable minimally invasive option when weighing efficacy and risks [281]. In endoscopic corpus callosotomy, Engel Class I–III outcomes were attained in 63.2% of pediatric patients, comparable to those of open procedures [283]. Complications include transient diabetes insipidus, hyponatremia, cranial nerve palsies, and vascular injury [281, 282].

The advantages of ES include visualization of deep‐seated structures, minimal cortical disruption, and reduced hospitalization time [280]. Unlike other minimally invasive techniques, ES provides direct visual feedback and allows real‐time resection or disconnection. This may facilitate more complete treatment of the EZ [281, 284]. Compared with open resection, endoscopic techniques cause less damage to functional neural pathways (e.g., the uncinate and inferior longitudinal fasciculi), thereby lowering the risk of language or memory deficits [279]. Additionally, ES demonstrates reduced blood loss and shorter operative times, which contribute to a lower average length of hospital stay [283]. Despite these advantages, ES has some limitations and risks. The procedure has a steep learning curve, which requires proficiency in endoscopic anatomy and bimanual instrument control [280]. Restricted instrument mobility and narrow working angles may limit the extent of resection in complex cases. The need for specialized equipment may restrict accessibility in resource‐limited settings [283]. Multicenter randomized trials are needed to establish standardized protocols and evaluate long‐term outcomes across endoscopic procedures [281, 283].

Future directions for endoscopic epilepsy surgery include the robotic‐assisted platforms and advanced imaging for real‐time navigation [280]. The integration of smartphone‐based endoscopic systems may improve portability and intraoperative flexibility while reducing dependence on bulky video systems [285]. Additionally, efforts to reduce costs and enhance training simulators will be essential for broader adoption.

#### 4.2.5 Robot‐Assisted Techniques in Epilepsy Surgery

Robot‐assisted techniques have transformed epilepsy surgery by improving the precision, efficiency, and safety of neurosurgical procedures. These systems combine preoperative MRI or CT scans with real‐time stereotactic navigation using robotic arms such as robotized stereotactic assistance, NeuroMate, and AutoGuide. They achieve submillimeter accuracy in trajectory planning and instrument positioning. Registration is performed via optical laser tracking or fiducial markers. This process integrates preoperative vascular and structural images with the intraoperative anatomy. As a result, surgical trajectories can be designed to avoid critical blood vessels and functional brain areas [286, 287, 288].

Multiple clinical studies have established the accuracy, efficacy, and safety of robot‐assisted systems in epilepsy surgery. These systems are primarily applied in three clinical areas: (1) SEEG electrode implantation: Robot‐assisted platforms enable SEEG electrode placement with submillimeter precision [286, 289]. A systematic review by Vasconcellos et al. of 855 patients across 29 studies reported a mean target point error of 2.13 mm [290]. Compared with manual techniques, robot‐assisted SEEG significantly reduced entry point error [291]. Meta‐analyses indicate complication rates of 5.1%–7.7% for surgical site infection, with symptomatic hemorrhage occurring in only 0.2% of cases [292]. (2) LITT: MRI‐guided robotic systems position laser fibers for EZ ablation and disconnective procedures [293, 294]. A meta‐analysis of 11 studies found that robot‐assisted LITT offers targeting precision comparable to non‐robotic methods, with a mean localization error of 1.66 mm [250]. Seizure‐free outcomes were achieved in 58% of patients and 86% experienced improvement. No significant differences were observed in seizure freedom, improvement, or complication rates between robotic and non‐robotic LITT [250]. (3) RFTC: Robot‐assisted stereotactic systems allow precise implantation of SEEG electrodes, which can subsequently guide RFTC [295]. Wu et al. used the robot‐assisted stereotactic system to implant depth electrodes bilaterally in the mesial temporal lobes for EZ identification and RFTC delivery [296]. The procedure led to significant seizure reduction, with 57% of patients reaching Engel Class I outcome at a mean follow‐up of 44.6 months. No permanent neurological deficits were reported, supporting the safety of this robotic method [296].

Compared with manual techniques, robot‐assisted systems enhanced accuracy, efficiency, and versatility of surgery. First, robotic guidance improves entry point accuracy by 60% and target point accuracy by over 30% compared to manual methods [287]. These systems help lower the risk of targeting mistakes and reduce surgeon fatigue. This makes surgeries safer, even during long procedures [286, 289]. Second, robotic assistance shortens operative time. For SEEG electrode implantation, robot‐assisted systems reduce total procedure time by 23.66 min and decrease implantation time per electrode by 3.35 min [291, 297]. Third, modular designs support interchangeable instruments, enabling multimodal treatments [298]. Despite these technical advantages, the adoption of robotic systems faces challenges. High initial costs, a steep learning curve, and specialized maintenance limit accessibility [289, 298].

Future developments aim to integrate AI, miniaturize instruments, refine training, and lower costs. Machine learning models trained on SEEG data may help automate ablation during LITT [299]. Miniaturized magnetic tools can improve navigation in endoscopic procedures [300]. Standardized training programs using three‐dimensional‐printed skull models are essential for skill acquisition [299, 301]. Economic measures such as disposable instruments may help reduce costs [299].

#### 4.2.6 Closed‐Loop Responsive Surgery System

Closed‐loop responsive surgery systems are advanced implantable devices. They continuously track neural activity and administer targeted electrical stimulation upon detecting abnormal patterns. Currently, the RNS system is the only RNS device approved for clinical use [302]. It uses chronically implanted electrodes linked to a neurostimulator that constantly analyzes electrocorticographic signals [302]. Detection algorithms identify epileptiform discharges, and responsive stimulation is delivered via electrodes placed at predefined EZ [302, 303]. The fundamental principle involves real‐time signal processing, detection of pathological activity, and delivery of electrical pulses within milliseconds. This forms an automated feedback loop that helps prevent seizures [304, 305]. The therapeutic mechanism includes both immediate seizure suppression through direct stimulation and long‐term neuromodulation via network plasticity changes [303, 306]. Clinical applications and therapeutic efficacy are discussed in the next section.

Recent advances have expanded closed‐loop functions beyond basic responsive stimulation. For example, the closed‐loop system for electrical stimulation platform serves as an important research tool that supports complex closed‐loop paradigms with multiple detection features and stimulation parameters [305]. It offers several operation modes, including real‐time feature detection and state estimation models, enabling researchers to apply complex intervention strategies [305]. Technological progress has also promoted the development of bimodal systems that integrate electrical stimulation with drug delivery. One such system is a bimodal closed‐loop neurostimulation implant that provides both electrical stimulation and on‐demand drug release [304]. It uses ultra‐flexible neural probes coated with platinum–iridium, which improve signal quality and stimulation effectiveness while reducing tissue injury [304].

In conclusion, minimally invasive techniques represent a safe and effective treatment for DRE. Although these methods are less effective than resective surgery in achieving complete seizure freedom, they offer significant advantages in reduced invasiveness and cognitive preservation [144]. Continued technical refinements are expected to further increase their clinical value, such as robot‐assisted system [297]. However, challenges such as limited access and procedure‐related risks persist. Complementary approaches such as neurostimulation offer additional options for modulating neural activity without resection.

### 4.3 Breaking the Seizure Network: Neurostimulation Therapy

In contrast to ASMs and surgical interventions, neuromodulation interrupts the seizure network by delivering targeted stimuli to specific brain regions. Three neurostimulation techniques have been approved for DRE management: vagus nerve stimulation (VNS), deep brain stimulation of the anterior nucleus of the thalamus (ANT‐DBS), and RNS [15]. However, these modalities require permanent implantation, which may be undesirable for some patients. Recent theoretical and technological advances have driven the development of noninvasive neurostimulation techniques, which show considerable potential for clinical application.

Neurostimulation offers several advantages, including improved tolerability and reversibility. Compared to ASMs, neurostimulation therapies demonstrate superior tolerability by minimizing the risk of drug toxicity. Unlike surgery, which results in permanent resection, neurostimulation can be adjusted or discontinued without causing permanent alterations to brain structure or function. Additionally, some neurostimulation therapies may enhance cognitive and emotional outcomes in patients.

#### 4.3.1 Invasive Neurostimulation

**FIGURE 3:**
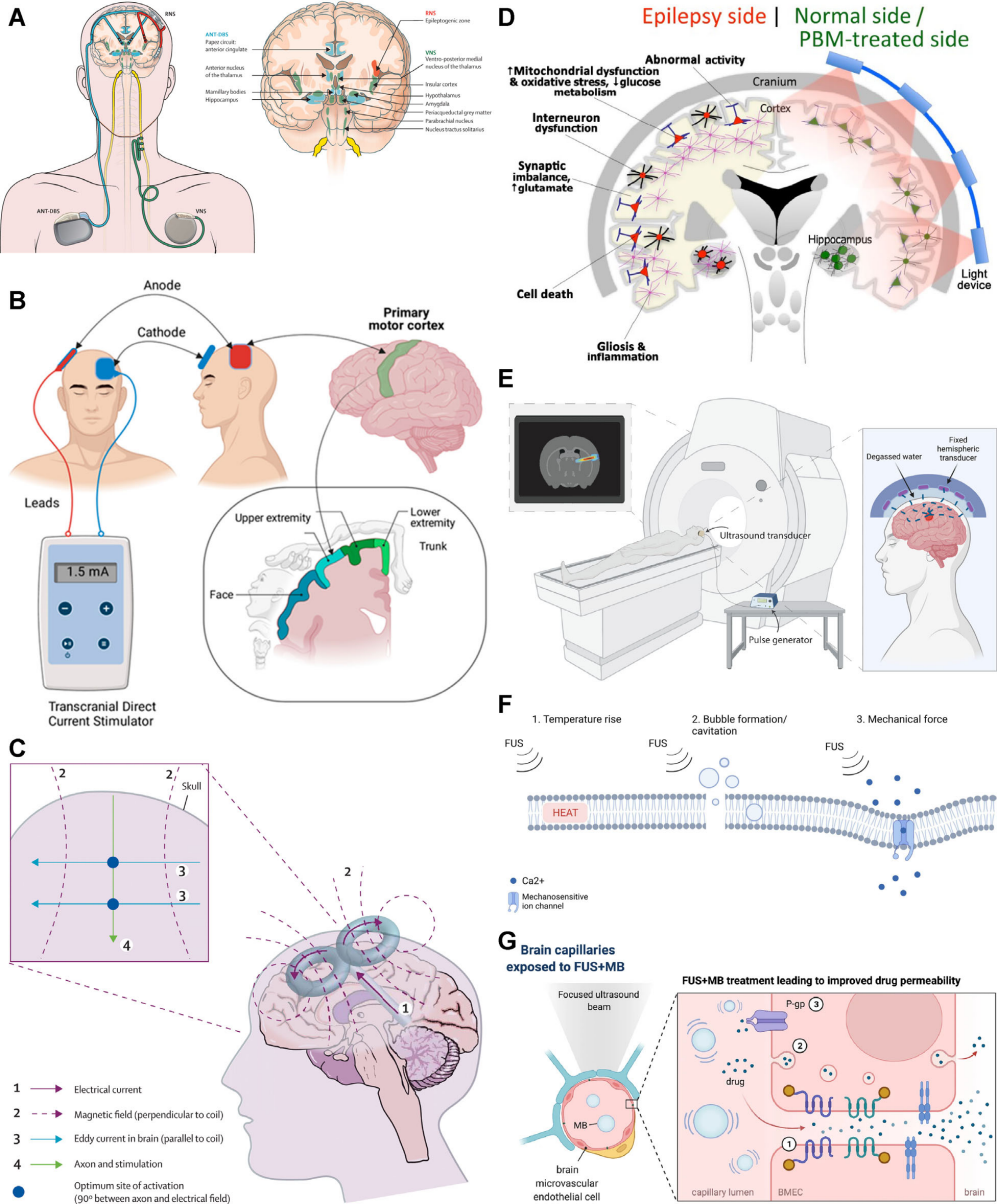
Neurostimulation and FUS in epilepsy treatment. (A) The schematic diagram of VNS, ANT‐DBS, and RNS. Reprinted with permission from Ref. [15]. Copyright 2021 Elsevier. (B–D) Noninvasive neuromodulation techniques used in epilepsy treatment include tDCS, TMS, and PBM. Reprinted with permission from Ref. [308]. Copyright 2022 MDPI. Reprinted with permission from Ref. [309]. Copyright 2008 Elsevier. Reprinted with permission from Ref. [310]. Copyright 2023 Wolters Kluwer. (E) FUS exerts biological effects through mechanical energy generated by ultrasound beams, which can be monitored using magnetic resonance. Adapted with permission from Ref. [311, 312]. Copyright 2023 MDPI. Copyright 2022 Open Access Government. (F) LIFUS deforms mechanically sensitive ion channels, causing ion influx and channel opening, thereby modulating action potentials and neural conduction. Reprinted with permission from Ref. [313]. Copyright 2022 Frontiers. (G) LIFUS induces microbubble oscillation (following intravenous administration), temporarily opening the BBB. Adapted with permission from Ref. [314]. Copyright 2022 Springer. ANT‐DBS, deep brain stimulation of the anterior nucleus of the thalamus; FUS, focused ultrasound; PBM, photobiomodulation; RNS, responsive neurostimulation; VNS, vagus nerve stimulation.

VNS modulates neural activity by implanting electrodes on the left side of the neck. VNS works through a dual mechanism of acute electrical stimulation and chronic neural regulation. It triggers various neurophysiological changes by affecting neural pathways in areas such as the brainstem, thalamus, and cortex. Acute stimulation can rapidly alter neural electrical activity and suppress abnormal synchronous discharges. Chronic regulation reshapes neural circuit function and maintains antiepileptic effects by regulating GABA function and inhibiting neuroinflammation [15] (Figure 3A). ANT‐DBS modulates abnormal neural activity by delivering pulsed electrical currents to the anterior thalamic nucleus, which is linked to its involvement in the Papez circuit [15] (Figure 3A). RNS incorporates a stimulator surgically implanted in the cranium, along with two depth electrodes or subdural strips positioned intracranially close to seizure sites, continuously monitoring electrocorticographic signals and providing customizable stimulation [307] (Figure 3A). The clinical trials involving invasive neurostimulation therapies are detailed in Table 2.

Clinical evidence has well supported the efficacy of invasive neurostimulation to treat epilepsy. Two comprehensive systematic reviews reported long‐term response rates of approximately 50% (56%–63%) for both adults and children treated with VNS, whereas seizure freedom rates ranged from 8% to 12% [315, 316]. Additionally, a meta‐analysis indicated that individuals receiving high‐level VNS were 1.73 times more likely to achieve a 50% reduction in seizures compared to those receiving low‐level stimulation. Common adverse effects of VNS include vocal cord paralysis, pain, and coughing [191]. Clinical studies report that left vocal cord paralysis accounts for approximately 1.5% of all complications related to VNS. Most cases are mild and transient. Symptoms often include hoarseness or cough during stimulation. These effects may result from surgical trauma or chronic denervation caused by stimulation [317, 318]. The condition usually resolves spontaneously or after adjustment of stimulation parameters.

A systematic review of 25 clinical trials found a short‐term responder rate of 67% and a long‐term responder rate of 72% based on a per‐protocol analysis among patients with DRE undergoing ANT‐DBS therapy [319]. Predictive factors for treatment response include electrode placement and seizure types [320]. The most frequent adverse effects of ANT‐DBS are related to implantation site infections, pain, and emotional disturbances [228, 230]. The emotional disturbances include depression (37.3%, mostly in patients with prior history), suicidal ideation (10.0%), and memory impairment (27.3%). Notably, neuropsychological outcomes generally improved over time [228, 230].

A double‐blind, sham‐controlled, parallel‐group randomized trial assessed the efficacy of RNS in patients with DRE. The RNS group demonstrated a significant reduction in seizure frequency compared to the sham group (−37.9% vs. −17.3%, *p* = 0.012) during the blinded phase. This effect persisted throughout follow‐up, with an average seizure reduction of 53% after 2 years [232]. The most common adverse event associated with RNS is infection [234, 236]. The system effectively addresses various epilepsy types, including neocortical epilepsy and idiopathic generalized epilepsy, though response patterns vary based on EZ localization [306, 321].

Patients undergoing surgery for other diseases while implanted with neurostimulation devices present distinct perioperative management challenges and risks. For instance, VNS can activate the recurrent laryngeal nerve, resulting in vocal fold adduction and laryngeal spasms. Under general anesthesia, patients are unable to report discomfort, which increases the likelihood of upper airway obstruction and aspiration due to reflux [322]. Additionally, during device testing or intraoperative stimulation, VNS may induce strong vagal responses, causing severe bradycardia and hypotension [322].

Despite demonstrated efficacy and favorable safety profiles of VNS, ANT‐DBS, and RNS, their widespread clinical adoption remains limited by several challenges: (1) the invasiveness of device implantation necessitates invasive surgical procedures and lifelong device maintenance, which may deter patient eligibility and compliance; (2) substantial financial burdens related to device costs, surgery, and follow‐up care; and (3) heterogeneous and often unpredictable treatment responses, with a proportion of patients failing to achieve adequate seizure control [15].

#### 4.3.2 Noninvasive Neurostimulation

Despite the notable success of VNS, ANT‐DBS, and RNS, their clinical application remains constrained by their invasive nature, associated adverse events, and variable outcomes. Neurostimulation devices necessitate surgical procedures for electrode placement, pulse generator installation, and periodic battery replacements, increasing perioperative risk for patients. In response, emerging noninvasive brain stimulation techniques, such as transcranial direct current stimulation (tDCS), repetitive transcranial magnetic stimulation (rTMS), and photobiomodulation (PBM), offer potential alternatives for patients seeking safer, less invasive treatment options.

tDCS modulates cortical neuron excitability through low‐intensity direct current stimulation [308] (Figure 3B). rTMS, which regulates brain activity via magnetic currents to decrease seizure susceptibility, has also emerged as a potential noninvasive treatment [309] (Figure 3C). PBM uses red to near‐infrared (NIR) light (*λ* = 600–1000 nm) on body tissues to regulate neuronal activity and survival, making it an ideal treatment option for epilepsy [310]. PBM is noninvasive, and the device is easy to use. The mechanism of this treatment involves enhancing ATP production, activating neuroprotective genes, and reducing neuroinflammation [310] (Figure 3D).

Clinical trials suggest that tDCS may reduce seizure frequency in patients with DRE [323]. The systematic review involves 62 independent studies, including 331 participants (15 healthy subjects and 316 epilepsy patients). The frequency and duration of stimuli are critical factors in determining the therapeutic outcome (*n* = 70) [324]. Additionally, the frequency of stimuli repetitions and the duration of intervals between stimuli play a critical role in determining therapeutic effectiveness [324, 325]. Following safety evaluations, no severe adverse reactions were reported in patients with DRE undergoing tDCS treatment.

Several studies have documented a notably elevated efficacy of rTMS in treating epilepsy, whereas others have indicated that rTMS may not effectively diminish seizure frequency in patients with DRE [326, 327, 328]. Due to the limited evidence and small sample size in existing clinical studies, the efficacy of rTMS in the treatment of focal DRE remains uncertain [329]. As a result, it is important for upcoming studies to have sufficient length and participants in order to evaluate the lasting effects of rTMS therapy, such as decreased seizures, enhanced quality of life, and possible negative impacts.

Clinical studies on PBM treatment for epilepsy are still limited. However, transcranial PBM helmets have been successfully used in patients with Alzheimer's disease and Parkinson's disease, demonstrating their feasibility in human subjects [330, 331]. The standard method involves transcranial application using helmets or panels equipped with light‐emitting diodes or low‐level lasers. The device is placed on the scalp, targeting the frontal cortex, cerebral cortex, and other brain regions noninvasively [332]. The preliminary human studies suggest that PBM can lead to measurable improvements in cognitive function, memory, and behavioral symptoms in patients with neurological conditions [310, 330, 331, 332]. It has an impeccable safety record, with almost no or no evidence of adverse effects or toxicity to body cells [333].

Several limitations hinder the broader adoption of noninvasive neurostimulation in clinical practice. Chief among these are the significant variations in sample sizes, methodologies, and stimulation protocols across studies. Future research must address these inconsistencies with adequately powered studies that assess long‐term outcomes. Additionally, although noninvasive techniques effectively modulate superficial brain networks, targeting deeper brain structures remains a formidable challenge. Although neurostimulation techniques provide reversible and adjustable therapeutic options, their invasive nature and cost limit broad application. Emerging technologies such as FUS offer promising noninvasive alternatives for precise neuromodulation or ablation.

#### 4.3.3 Preclinical Evidence of Invasive Neurostimulation

Preliminary preclinical investigations have illustrated the potential of noninvasive neurostimulation in reducing epileptic seizures in animal models. Preclinical studies demonstrated that long‐term tDCS administration can mitigate seizures and ameliorate pathology in a rat pilocarpine model [334]. The application of tDCS in rats with chronic spontaneous seizures resulted in fewer interictal spikes and an increase in delta oscillations, whereas beta and gamma oscillations decreased [335]. TDCS therapy has also been shown to improve cognitive deficits following SE [334]. Furthermore, the anticonvulsive effect is specific to cathode stimulation, as anodal stimulation did not show any impact on seizure activity [336]. Low‐frequency rTMS has suppressive effects on electrical activity in epileptic rats [337]. Long term use of rTMS can lead to changes in seizure threshold. The seizures of epileptic rats treated with rTMS were significantly less than those of sham rTMS animals [338]. Additionally, several studies have investigated the effects of PBM in animal models of epilepsy. In a pentylenetetrazole‐induced rat model, PBM significantly reduced seizure frequency, lowered mortality rates, and improved pathological outcomes [339].

### 4.4 The Therapeutic Potential of FUS in Epilepsy: Neuromodulation and Tissue Ablation

FUS has emerged as a noninvasive technology capable of both neurostimulation and targeted tissue ablation. FUS uses ultrasound beams to modulate the structure or function of targeted tissues via mechanical energy [17]. By adjusting FUS parameters, such as intensity, frequency, and duration, various biological effects can be achieved, including neuromodulation, tissue ablation, and BBB permeabilization [17].

The key advantages of FUS for epilepsy treatment are its noninvasive characteristic, ability to target deep brain structures, and precise localization capabilities. Moreover, magnetic resonance‐guided focused ultrasound (MRgFUS) offers high‐resolution, real‐time imaging for accurately targeting specific tissues. MRI thermographic guidance further ensures controlled temperature and energy delivery, minimizing damage to surrounding nontarget areas [17] (Figure 3E).

#### 4.4.1 Low‐Intensity Focused Ultrasound (LIFUS): Regulating Seizure Network

LIFUS (<100 W/cm^2^) exerts its neuromodulatory effects by deforming mechanically sensitive ion channels in cell membranes, facilitating ion influx and channel opening, which ultimately triggers action potentials and neural conduction [313] (Figure 3F). Importantly, LIFUS does not cause thermal injury or permanent anatomical changes, making it an increasingly recognized nonsurgical therapeutic method for neuromodulation. Computational modeling studies have demonstrated that the intracranial temperature increase induced by LIFUS can be as low as 0.009°C, a level insufficient to cause thermal damage [340]. Furthermore, recent Phase 1 clinical trials involving stroke patients have verified that even at stimulation intensities up to 8 W/cm^2^, no new lesions or thermal injuries, such as skin burns necessitating medical intervention, were detected on MRI [341]. Additionally, studies involving patients with depression have confirmed that the modulatory effects of LIFUS on deep brain regions are reversible [342].

To date, only one pilot study has investigated LIFUS in patients with DRE. In this study, conducted on six patients with DRE, two experienced reduced seizure frequency after treatment, and changes in stereoelectroencephalography power were observed. Adverse events included scalp warming and temporary difficulties with naming and memory. However, the short follow‐up period (72 h) limits the scope of this study's findings [343].

#### 4.4.2 High‐Intensity Focused Ultrasound (HIFUS): Ablating Epileptic Tissue

HIFUS (>200 W/cm^2^) provides a noninvasive alternative to resection surgery by using ultrasound waves to achieve thermal ablation of targeted brain tissue. Compared to traditional surgical methods, HIFUS allows real‐time monitoring through a triple feedback mechanism: immediate clinical feedback, live thermography, and anatomical imaging to visualize the lesion [17]. This feature is one of the key advantages of HIFUS technology. Several case reports and pilot studies have been published on HIFUS applications.

In 2020, the first case report of transcranial MRgFUS for mesial TLE was published, describing 12 sonication sessions targeting the patient's left hippocampus, which led to almost complete seizure freedom for 12 months [344]. In a more recent Phase 1 open‐label trial, two patients with refractory focal epilepsy underwent anterior nucleus of the thalamus FUS ablation, both of whom experienced significant seizure reduction, with one achieving complete seizure freedom at 12 months [238]. However, a potential limitation of MRgFUS procedures is the risk of increased skull heating, which remains a consideration in clinical applications [17].

At present, clinical reports on the use of HIFUS for DRE are based on individual cases or small‐scale preliminary studies [144]. This is mainly due to the challenges associated with the application of this technology in neurosurgery, particularly the need to penetrate the skull barrier and the exploration of precise ablation targets for epilepsy [345]. It is necessary to focus on a limited number of patients during the initial phases of rigorous investigation. However, it does not mean that the application potential of HIFUS is limited. On the contrary, HIFUS has achieved success in the treatment of neurological diseases such as essential tremor and Parkinson's disease [346, 347]. These diseases have been studied with larger sample sizes and have even received clinical certification in certain regions [348]. Therefore, the application of HIFUS in epilepsy treatment is constantly consolidating. Skull heating is a crucial safety consideration for HIFUS and requires further evaluation. With the popularization of technology and the accumulation of clinical experience, its applicable patient population is expected to rapidly expand.

#### 4.4.3 FUS Provides Opportunities and Challenges in Delivering Biologics for Epilepsy

FUS is being actively explored for its ability to transiently open the BBB. By inducing the oscillation of intravenously administered microbubbles, FUS temporarily disrupts the tight junctions between capillary endothelial cells, allowing for localized BBB permeability at the targeted site [349] (Figure 3G). This technique offers a promising avenue for advancing drug delivery strategies in epilepsy treatment.

#### 4.4.4 Preclinial Evidence

Preliminary preclinical studies support the efficacy of LIFUS in modulating seizure networks. In rodent epilepsy models, LIFUS reduced both acute seizure activity and spontaneous recurrent seizures (SRSs) without causing damage to brain tissue [350]. This approach also mitigated gliosis, neuroinflammation, and hippocampal volume loss [351]. Furthermore, recent findings suggest that the effects of LIFUS on epileptiform activities are highly parameter‐dependent. For example, a stimulation protocol with elongated bursts and sufficient intervals effectively suppressed epileptiform activities, whereas the same burst duration with shorter intervals exacerbated them. This parameter‐dependent modulation is likely mediated by changes in glutamate and GABA levels [352].

Initial preclinical studies have yielded promising results. The combination of MRgFUS and intravenous microbubbles efficiently opened the BBB in a rat pilocarpine seizure model, facilitating the entry of neurotoxin quinolinic acid—ordinarily unable to cross the BBB—into the hippocampus, where it selectively damaged neurons. This approach resulted in a reduction in seizure frequency, with two rats experiencing a complete absence of convulsive seizures [353].

The potential of FUS in epilepsy treatment is evident, as summarized in Table 2, which outlines relevant clinical trials. Table 3 further compares key features of ASMs, neurostimulation, surgery, and FUS. However, the safety and long‐term efficacy of FUS in epilepsy have yet to be fully assessed. Ongoing clinical trials with larger cohorts and extended follow‐up periods aim to establish standardized parameters for ultrasound‐based treatments, provided no major safety concerns emerge. FUS also facilitates targeted drug delivery across the BBB, introducing new possibilities for biologic and nanomaterial‐based therapies.

**TABLE 3:** The main features of antiseizure medications (ASMs), neurostimulation, surgery, and focused ultrasound (FUS).

	ASMs	VNS	ANT‐DBS	RNS	Surgery	LIFUS	HIFUS
Therapeutic target	Epileptogenic focus	Vagus nerve	Anterior nucleus of the thalamus	Epileptogenic focus	Epileptogenic focus	Epileptogenic focus	Epileptogenic focus
Mechanism	Regulating neuronal membrane potential, ion channels, and neurotransmitter release	Stimulation of the ascending projection; regulating neurotransmitters	Regulating the Papez circuit	High frequency electrical stimulation interrupts the seizure network	Excision of epileptic foci	Deformation of mechanically sensitive ion channels in cell membranes	Thermal ablation of epileptic foci
Types of treatment	Palliative	Palliative	Palliative	Palliative	Curative	Palliative	Curative
Indications	All	DRE with not localized, multifocal, or not resectable focus	DRE with bitemporal, multi lesion, or nonlocal focus	DRE with bitemporal or eloquent focus	DRE with resectable single focus	DRE, but the specific details are still unclear	DRE, but the specific details are still unclear
Invasive	—	Middle	Middle	Middle	High	Low	Low
Anatomical changes	No	No	No	No	Yes	No	Yes
Cost	Low	High	High	High	High	Unclear	Unclear
Compliance	Moderate	High	High	High	High	Unclear	Unclear
Adverse effects	Dizziness, nausea, allergic reactions, sexual dysfunction, etc.	Infection, dyspnea, vocal cord paralysis, pain, etc.	Implantation site infection, pain, and emotional disorders	Infection	Infection, bleeding, neurological damage, etc.	Skull heating	Skull heating

Abbreviations: ANT‐DBS, deep brain stimulation of the anterior nucleus of the thalamus; ASMs, anti‐seizure medications; DRE, drug‐resistant epilepsy; FUS, focused ultrasound; HIFUS, high‐intensity focused ultrasound; LIFUS, low‐intensity focused ultrasound; RNS, responsive neurostimulation; VNS, vagus nerve stimulation.

### 4.5 Nanomaterials as a Promising Therapeutic Potential for Epilepsy

Nano‐delivery systems offer innovative solutions to overcome the limitations of conventional ASMs, particularly in bypassing the BBB and enabling targeted delivery [18]. It is important to note that uncertainties remain surrounding optimal dosage in clinical translation. Moreover, the integration of nanomaterials into neurostimulation technologies has the potential to improve both their safety and effectiveness, providing new therapeutic options for patients [20]. It is important to note that many of the nanomaterial‐based strategies discussed in this section, while promising, are currently primarily supported by preclinical evidence from animal models. Their translational potential and clinical efficacy in humans remain to be fully established in future clinical trials.

The advantages of nano‐delivery systems include improved bioavailability, targeted delivery, and reduced toxicity. Unlike invasive methods such as intrathecal or intraventricular delivery, which bypass the BBB, nano‐delivery systems provide a noninvasive alternative [18]. The integration of nanomaterials into neural interfaces enhances electrode functionality, enabling interaction with both the electrophysiological and biochemical aspects of the nervous system. This approach offers innovative strategies for controlling epileptic seizures [354].

#### 4.5.1 Nano‐Delivery Systems for Epilepsy Treatment

**FIGURE 4:**
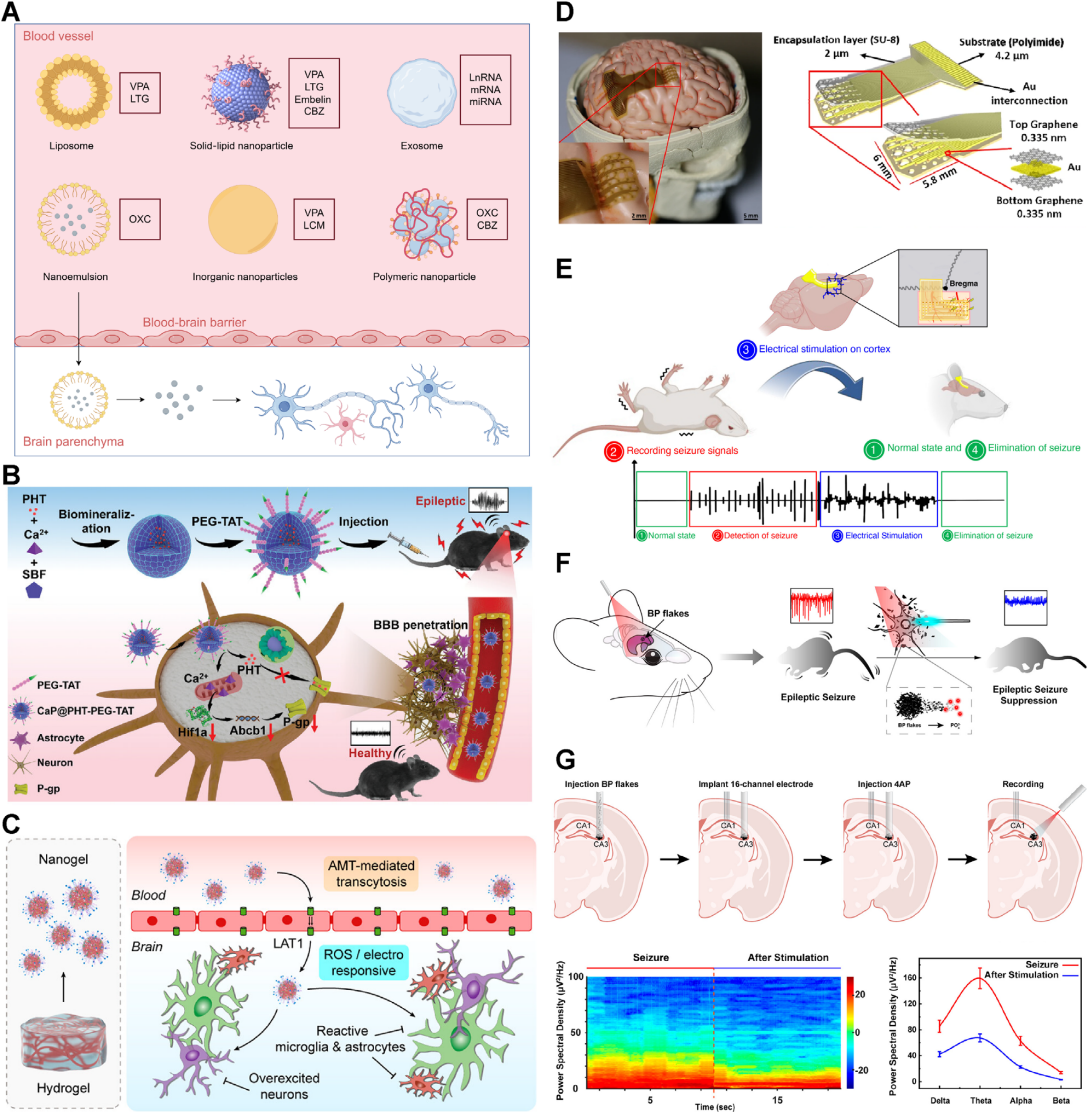
Nanomaterials in epilepsy treatment. (A) Various nanocarriers facilitate ASMs’ passage through the BBB. Adapted with permission from Ref. [18]. Copyright 2022 John Wiley & Sons. The image is drawn by Figdraw. (B) The nanoformulation eliminates phenytoin resistance in epileptic neurons while reducing seizures. Reprinted with permission from Ref. [356]. Copyright 2023 John Wiley & Sons. (C) Dual‐reactive nanogel targeting epileptic foci to remodel aberrant circuits and inflammatory microenvironments. Reprinted with permission from Ref. [19]. Copyright 2023 American Chemical Society. (D) Schematic illustration of graphene electrode array fabrication. Reprinted with permission from Ref. [357]. Copyright 2023 Springer. (E) A graphene electrode array implanted into the cortex can detect and modulate epileptic discharges in real time. Reprinted with permission from Ref. [20]. Copyright 2024 Elsevier. (F) Illustration of BP flake‐assisted neuromodulation for inhibiting epileptic electrical signals. Adapted with permission from Ref. [358]. Copyright 2024 American Chemical Society. (G) Epileptic signals are inhibited by BP flake‐enabled NIR neuromodulation. Adapted with permission from Ref. [358]. Copyright 2024 American Chemical Society. BBB, blood–brain barrier; CBZ, carbamazepine; LCM, lacosamide; lncRNA, long non‐coding RNA; LTG, lamotrigine; miRNA, microRNA; mRNA, messenger RNA; OLZ, olanzapine; OXC, oxcarbazepine; ROS, reactive oxygen species; VPA, valproic acid.

Alterations in the BBB play a critical role in the pathophysiology of epilepsy, influencing the absorption and therapeutic efficacy of ASMs. Research has demonstrated that the overexpression of drug efflux transporters at the BBB can impede the entry of ASMs into the brain, thereby inducing drug resistance [355]. Nanocarriers help transport drugs across the BBB through mechanisms like receptor‐mediated endocytosis, which raises ASM levels in the brain. Several nano‐delivery systems have been explored in preclinical studies of epilepsy, including inorganic nanoparticles, liposomes, polymer nanoparticles, solid lipid nanoparticles, and nanoemulsion formulations (Figure 4A) [18]. Table 4 compares traditional ASM delivery methods with nanocarrier systems.

**TABLE 4:** Comparison of the main features of antiseizure medications (ASMs) and nano‐delivery system.

Features	Anti‐seizure medications	Nano‐delivery system
Bioavailability	Low	High
Brain targeting	No	Yes
Stability	Low	High
Drug delivery route	Oral/Injection	Oral/Injection/Inhalation
Peripheral adverse effects	More	Less
Cost	Low	High
Level of accessibility	High	Low

#### 4.5.2 Nanomaterials for the Neurostimulation Therapy

Using nanotechnology might open the possibility of developing advanced functionalized nanomaterials that not only facilitate drug delivery but also enable the modulation of neural activity. Traditional neurostimulation therapies rely on rigid electrodes, which are mechanically invasive and can cause inflammation and tissue damage with long‐term implantation [359]. In contrast, multifunctional nanoelectric transducers are nanoscale materials that convert external energy sources (e.g., light) into localized electrical or thermal signals. This capability allows for the targeted control of specific neuronal populations, offers minimal invasiveness, and enables wireless functionality [360]. Moreover, surface modification of nanomaterials can enhance the biocompatibility of electrodes, ensuring sustained electrical stimulation efficacy over extended periods [361]. Nanomaterials can serve as electrodes for both neural signal detection and electrotherapy, enabling the integration of epilepsy diagnosis and treatment.

#### 4.5.3 Preclinical Evidence

Preclinical evidence has well supported the efficacy of these nanomedicines in treating epilepsy. For instance, Guan et al. developed a calcium phosphate‐based brain‐targeted nanoformulation for phenytoin. By incorporating PEGylated TAT peptides, the formulation facilitated BBB penetration and reduced the expression of efflux transporters in animal models, thereby improving phenytoin retention in the mice brain. This approach may help overcome the drug resistance associated with phenytoin in epilepsy treatment (Figure 4B) [356]. Furthermore, nanoengineered drug delivery systems offer the advantage of controlled drug release, ensuring sustained therapeutic levels of ASMs in the brain [362]. For example, Wu et al. developed a nanoengineered drug delivery system with a dual‐control release mechanism. The system is electroresponsive, allowing drug release to be triggered by pathological epileptiform discharges. It also enables sustained release to maintain therapeutic drug levels, which is essential for treating prolonged seizures. In vitro tests demonstrated that the system released drugs rapidly (within 30 s) and repeatedly upon exposure to an electric field simulating epileptic activity. These findings were confirmed by in vivo microdialysis studies in pentylenetetrazole‐induced acute seizure animal models. The results showed that the concentration of phenytoin in the brain increased 3.7‐fold within 30 min after seizure onset and remained elevated for more than 2 h. This pattern aligns with the required therapeutic profile. Furthermore, in a severe pilocarpine‐induced SE model, the system significantly delayed the onset of generalized seizures and SE and reduced EEG power—using only one‐fifth of the standard drug dose [362]. Additionally, certain nanomaterials have been shown to possess antioxidant properties, which can effectively neutralize free radicals, thereby reducing neuroinflammation and oxidative stress. Nanocarriers designed with these materials may enhance the therapeutic efficacy of ASMs. For example, Zhou et al. developed hydrogel nanoparticles that specifically target epileptic foci to deliver phenytoin. Simultaneously, these nanogels reduce oxidative stress and inflammation within the microenvironment, promoting synergistic modulation of epileptic lesions (Figure 4C) [19].

Minimizing peripheral adverse effects is important in the treatment of DRE. Surface modification of nanocarriers through the addition of coatings and ligands can further enhance targeted delivery to the brain, reducing peripheral adverse effects [18]. For example, encapsulating oxcarbazepine in surface‐modified nanoparticles enhances its delivery to the brain while limiting placental permeability, thereby preventing fetal exposure and associated adverse effects. This strategy offers promising potential for the safe treatment of epilepsy in pregnant women [363].

Compared to traditional neurostimulation, the graphene electrode array shows more promising therapeutic outcomes, including reduced physical damage, minimal immune response, enhanced biocompatibility, and superior electrical conductivity. Lim et al. demonstrated the use of a graphene electrode array that facilitates high‐resolution detection of local field potentials from the cortical surface, while simultaneously providing electrical stimulation to alleviate epileptic seizures [357] (Figure 4D,E). In addition, nanomaterials possess broad‐spectrum light absorption properties, allowing NIR light to penetrate biological tissues. This capability supports optical neuromodulation using nanomaterials, overcoming the limitation of visible light's inability to effectively penetrate tissue, thus enabling wireless and remote neuromodulation. Yang et al. demonstrated the effectiveness of NIR neuromodulation using nanomaterials in a mouse model of epilepsy. Photothermal black phosphorus (BP) flakes, exfoliated into crystals, enhanced neural activity by modulating membrane capacitive currents in hippocampal neurons through NIR photothermal neuromodulation (Figure 4F,G). This approach successfully inhibited epileptic signals in the mouse epilepsy model, offering a low‐invasive, biocompatible alternative to conventional treatments [358].

Despite these advances, there remain significant challenges in employing nanomaterials for epilepsy treatment. Key hurdles include uncertainties surrounding optimal dosage, anatomical differences between animal models and humans, and limited data on how nanocarrier properties affect CNS biodistribution [364]. These factors complicate the translation of preclinical findings to clinical settings, posing obstacles to the successful implementation of nanotechnology‐based therapies for epilepsy.

### 4.6 Restoring Balance in Epilepsy: The Role of GABAergic Cell Therapy

**FIGURE 5:**
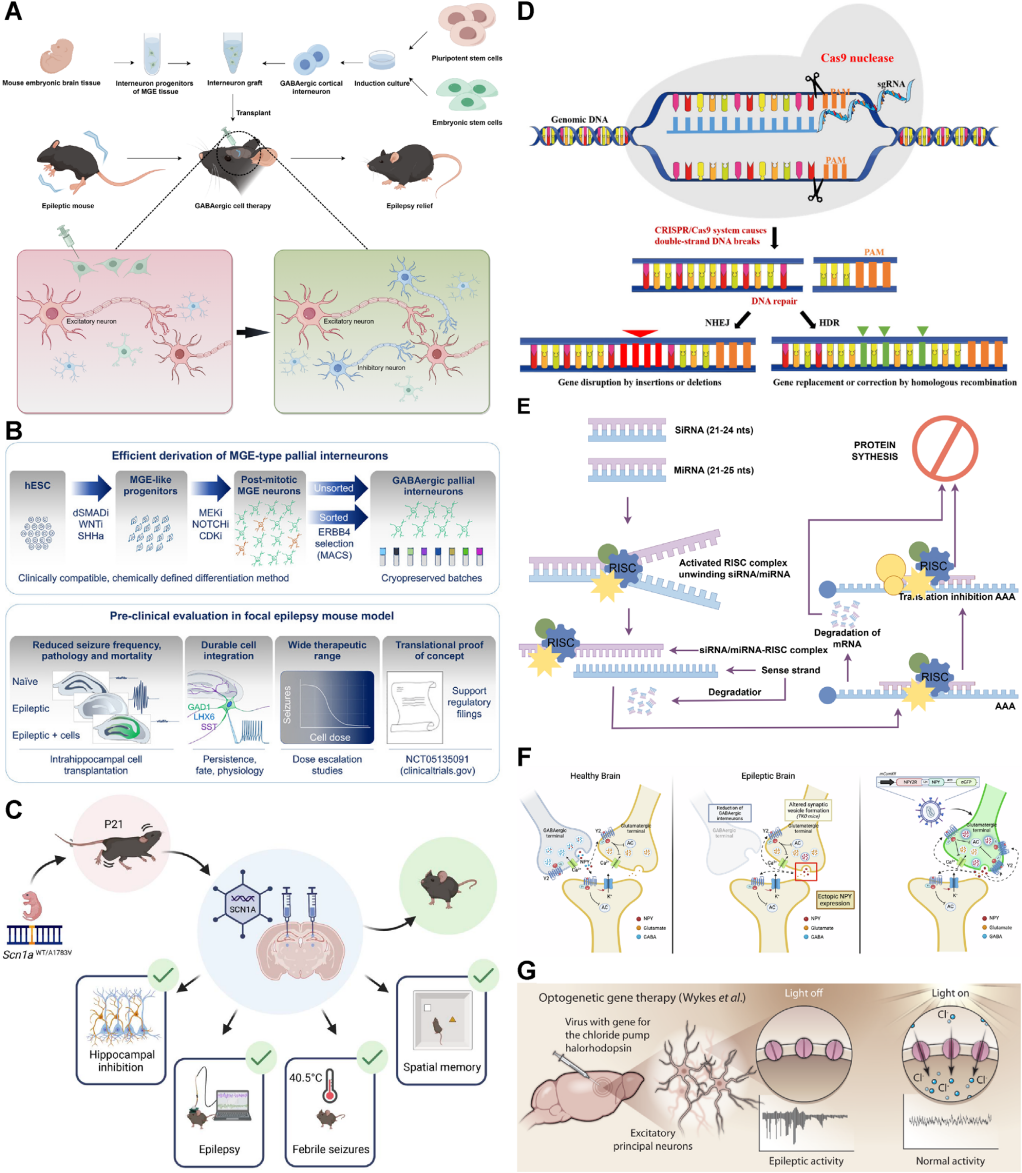
GABAergic cell therapy and gene therapy in epilepsy treatment. (A) Primary cells derived from mouse embryonic tissue or GABAergic cells induced from PSCs or ESCs are transplanted into the epileptic foci of epileptic mice. These cells differentiate into inhibitory neurons, helping control abnormal discharges at the epileptic foci. The figure is drawn by Figdraw. (B) Treatment of chronic focal epilepsy with human pallial MGE‐type GABAergic interneurons. Adapted with permission from Ref. [366]. Copyright 2023 Cell Press. (C) In mice with DS, viral vector‐mediated expression of Nav1.1 reduces seizure. Reprinted with permission from Ref. [367]. Copyright 2023 American Society for Clinical Investigation. (D) The working principle of CRISPR/Cas9 system. Reprinted with permission from Ref. [368]. Copyright 2022 MDPI. (E) The mechanism of action of RI. The figure is drawn by Figdraw. (F) Treatment of epilepsy with a gene therapy targeting neuropeptide Y and its receptor on dentate gyrus granule cells. Reprinted with permission from Ref. [369]. Copyright 2024 EMBOpress. (G) Optogenetics allowed chloride ions to enter neurons, reducing epileptic activity. Reprinted with permission from Ref. [370]. Copyright 2012 American Association for the Advance of Science. DNA, deoxyribonucleic acid; GABA, gamma‐aminobutyric acid; GAD, glutamate decarboxylase; MGE, medial ganglionic eminence.

The underlying pathophysiology of epilepsy is linked to the degeneration of GABAergic interneurons [365]. It is important to note that epilepsy is a heterogenous disorder with multiple etiological mechanisms; however, one well‐documented pathway involves the dysfunction of inhibitory interneurons [37]. Therefore, inhibitory cell transplantation could potentially restore the disrupted GABAergic balance in seizure onset zones, offering a means of addressing the underlying pathophysiology (Figure 5A).

Compared to other alternative treatments, GABAergic cell therapy holds a unique advantage in its capacity to reorganize neural networks and treat multi‐lesional epilepsy. Only a limited subset of patients with DRE are deemed suitable candidates for neurostimulation or surgery following thorough evaluation. Neurostimulation serves as a palliative measure that does not directly address the root cause of epilepsy. In contrast, GABAergic cell therapy shows promise in enhancing local synaptic inhibition, directly targeting the pathological processes of epilepsy [21]. For patients with diffuse or unresectable lesions, such as bilateral temporal lobe seizure foci, where surgery is not viable, GABAergic cell therapy may offer a potentially effective alternative.

#### 4.6.1 Medial Ganglionic Eminence (MGE) Progenitors

During embryonic development, interneurons from the MGE and caudal ganglionic eminence migrate to the cerebral cortex and forebrain, where they integrate into local circuits to provide inhibitory outputs [371]. Over 2 decades ago, researchers successfully transplanted MGE‐derived interneurons from embryonic mice into postnatal mice, where they migrated, survived, and produced GABA [372]. Hippocampal tissues from patients with epilepsy exhibit a reduction in MGE‐derived interneurons [365]. Furthermore, mutations in genes required for MGE interneuron generation and function have been identified in various developmental epileptic disorders [373]. Therefore, restoring the function of MGE interneurons could be a potential therapeutic strategy for epilepsy.

#### 4.6.2 GABAergic Progenitors From Stem Cells

Embryonic stem cells (ESCs) and induced pluripotent stem cells (iPSCs) offer an unlimited source of therapeutic cells [374, 375]. However, the ethical viability of ESCs continues to be widely debated, mainly because they require the destruction of human embryos for their isolation [376]. In contrast, iPSCs possess similar pluripotency to ESCs but are generally viewed as a less ethically contentious alternative as their generation does not involve the use of embryos [376].

#### 4.6.3 Preclinical Evidence

Preliminary preclinical studies have explored the therapeutic potential of MGE progenitor transplantation in improving seizures in animal models. MGE progenitor transplantation shows promise in epilepsy prevention, with preclinical data suggesting that early transplantation, prior to disease onset, increases seizure thresholds, reduces seizure severity, and lowers mortality rates [377]. Second, in chronic epilepsy models, MGE progenitor transplants significantly reduce the frequency and duration of SRSs [378]. Lastly, this therapeutic approach has also been shown to improve spatial learning and memory in models of chronic TLE [379].

Initial studies involved mouse ESCs (mESCs), where mESC‐derived neural progenitors were transplanted into the hippocampus of epileptic mice. These graft‐derived cells differentiated into functional GABAergic interneurons, exhibiting electrophysiological properties typical of interneurons in the hilus [374]. These findings have paved the way for further investigations. More recently, human ESCs (hESCs) and iPSCs (hiPSCs) have been employed in research (Figure 5B). Transplantation of hESC/hiPSC‐derived MGE‐like interneuron precursors into the hippocampus of chronic epilepsy mouse models resulted in significant seizure reduction, along with improvements in cognitive, memory, and mood deficits [375]. This suggests that human stem cell‐derived interneuron progenitors may hold considerable promise as a therapeutic approach for epilepsy.

Although preclinical studies have demonstrated the efficacy of cell therapy in suppressing epileptic seizures, some ongoing human clinical trials have not yet yielded results (Table 2). There are some limitations of cell therapy need to be overcome in the future. First, the long‐term effectiveness of these grafts in animal models tends to diminish over time, potentially due to GABA interneuron depletion or progression of the underlying pathology [380]. The longevity and sustained seizure management capacity of transplanted neurons must be further evaluated. Second, hESC/hiPSC‐derived progenitors may contain immature, proliferating cells, posing a risk of tumorigenesis upon transplantation. The rejection reaction of interindividual transplantation also complicates the use of cell therapy. Finally, scaling up the production of human intermediate neural progenitor cells for transplantation is a critical challenge. It is crucial to recognize that while cross‐species cell therapy represents a promising strategy to overcome donor cell shortages, it also raises substantial safety issues requiring rigorous evaluation. Key concerns include immune‐mediated rejection responses, the potential transmission of zoonotic pathogens, and ethical implications associated with introducing nonhuman biological materials into patients. Therefore, although preclinical studies in this field remain exploratory, any future clinical application must be predicated on resolving these safety and ethical challenges through robust preclinical data and adherence to stringent regulatory guidelines. For genetic epilepsy, gene therapy provides a more targeted strategy.

### 4.7 Personalized Treatment for Epilepsy: The Potential of Gene Therapy

Seventy to eighty percent of epilepsy cases have genetic origins, with numerous pathogenic variants linked to genetic epilepsies [22]. These mutations are frequently associated with DEEs, which often result in premature death and neurodevelopmental disorders [23]. Currently, patients with these conditions have limited treatment options, but gene therapy offers a promising avenue for targeted intervention. By modifying gene expression or function, gene therapy can directly address the molecular pathology underlying these disorders. Furthermore, gene therapy approaches may provide symptom relief for patients with DRE of unknown etiology [22].

The potential advantages of gene therapy for epilepsy include its ability for personalization and the possibility of achieving long‐term therapeutic benefits. By reducing the need for frequent and high‐dosage ASMs, gene therapy may mitigate the adverse effects commonly associated with these drugs [23]. Additionally, it allows for enhanced precision by tailoring treatment to specific genetic profiles. In cases of monogenic epilepsy, gene therapy offers the potential to correct the underlying genetic mutations, thereby restoring neurological function and potentially providing a path to a lasting cure.

#### 4.7.1 Gene Therapy for Treating Etiology

Various gene therapies have been explored in preclinical models to target the phenotypic causes of epilepsy, including gene delivery, gene editing, antisense oligonucleotides (ASO), and RNA interference (RI) [381]. Gene delivery uses viral vectors, such as adeno‐associated virus serotype 9 (AAV9), to introduce healthy gene copies to compensate for loss‐of‐function mutations [381] (Figure 5C). Gene editing technologies, such as CRISPR/Cas9, can precisely target and modify DNA sequences to regulate gene expression [368] (Figure 5D). ASOs are short, single‐stranded DNA or RNA molecules designed to modulate gene expression by targeting specific mRNA sequences [382]. ASOs have demonstrated efficacy in treating gain‐of‐function mutations. RI uses small interfering RNA (siRNA) or microRNA (miRNA) to silence specific genes (Figure 5E).

#### 4.7.2 Gene Therapy for Relieving Symptoms

The multifaceted nature of epilepsy, where most seizures do not arise from a single molecular abnormality, complicates treatment strategies. Gene therapy presents a promising solution by modulating dysfunctional neural networks, either through enhancing inhibitory molecules or reducing excitatory ones, potentially alleviating symptoms in patients with DRE [22] (Figure 5F). Furthermore, optogenetics and chemogenetics have demonstrated the ability to suppress epileptic activity by activating inhibitory neurons or silencing excitatory ones in rodent models [383, 384] (Figure 5G). Optogenetics and chemogenetics are widely used techniques for targeted neuromodulation in neuroscience research [385]. Optogenetics involves the genetic introduction of light‐sensitive proteins called opsins into specific populations of neurons. This technique controls neural activity with light, achieving millisecond precision [383]. Light is typically delivered through implanted optical fibers. In contrast, chemogenetics relies on engineered receptors, which are activated by inert ligands. Unlike optogenetics, chemogenetics does not need permanent implants and can modulate neurons for several hours. Its main limitation is a lower temporal precision [384].

#### 4.7.3 Preclinical Evidence

Preliminary preclinical evidence confirms the efficacy of gene therapy in treating epilepsy. One example is DS, a hereditary brain disorder characterized by severe seizures and cognitive, motor, and behavioral deficits. This condition is caused by haploinsufficiency of the *SCN1A* gene, which encodes the alpha subunit of the Nav1.1 [29]. Fadila et al. demonstrated that delivering a modified *SCN1A* gene into the brains of young mice with DS (SCN1AA1783V/WT) via viral vectors improved seizure outcomes [367] (Figure 5C). Similarly, Mora‐Jimenez et al. confirmed the beneficial effects of *SCN1A* gene delivery on motor function and cognitive impairment in DS mice [386]. Yamagata et al. showed that dead Cas9 (dCas9)‐mediated activation of the *SCN1A* gene effectively treated haploinsufficiency in DS mice, restoring Nav1.1 sodium channel expression. Administering the *SCN1A*‐dCas9 activation system to DS pups via adeno‐associated viruses restored interneuron firing and reduced febrile seizures [387]. The administration of sodium voltage‐gated channel alpha subunit 2 ASO in mutant mice reduced gene expression, leading to fewer seizures and extended survival [388]. ASOs also hold promise for increasing gene expression. The inclusion of poison exon 20N in *SCN1A* triggers nonsense‐mediated decay of the transcript, thereby suppressing functional gene expression. To counter this, ASOs are designed to block exon 20N splicing. This intervention effectively restores productive *SCN1A* mRNA levels and boosts the synthesis of the Nav1.1 sodium channel. Consequently, it markedly reduced seizures and prevented sudden death in a DS mouse model [389]. Preclinical studies also suggest that RI could alleviate symptoms in certain genetic epilepsies, such as dynamin‐1‐associated DEEs [390].

Preclinical studies also provide encouraging evidence for the application of gene therapy in symptom management. For example, neuropeptide Y (NPY), an endogenous neuromodulator with anticonvulsant properties, reduces presynaptic glutamate release. Elevating NPY levels in the hippocampus via an AAV vector has been shown to significantly decrease SRSs in a mouse model of epilepsy [369] (Figure 5F). In a mouse model of acute epilepsy, temporally precise optogenetic silencing of hypothalamic orexin neurons during the pre‐seizure phase significantly reduced both the electrophysiological and behavioral severity of subsequent seizures [383]. The chemogenetic platform also shows therapeutic potential for epilepsy. The inhibitory channel BARNI (bradanicline‐ and acetylcholine‐activated receptor for neuronal inhibition), a designer receptor combining the α7 nAChR ligand‐binding domain with the glycine receptor pore, is activated by bradanicline. In mouse models of TLE, its activation demonstrated that BARNI effectively raises the seizure threshold and diminishes the frequency of spontaneous seizures for multiple hours [384].

Despite its promise, gene therapy faces significant challenges in translating from preclinical research to clinical application. First, the physiological differences between animal models and humans may lead to treatments that are effective in animal experiments failing in clinical trials. Therefore, developing more humanized models such as brain organoids derived from hiPSCs may help improve the clinical translation efficiency of gene therapy [391]. Additionally, gene therapy delivery systems must be cell‐specific to minimize adverse effects and toxicity in nontarget cells [392]. Lastly, identifying the optimal therapeutic window is essential, as DEEs often emerge in childhood, highlighting the need for early intervention to maximize treatment efficacy [393]. There are some ongoing clinical studies evaluating the efficacy and safety of gene therapy for DRE patients (Table 2). Through continuous technological innovation and research, it is expected that gene therapy will achieve clinical application in epilepsy treatment in the future.

## 5 Conclusion and Future Perspectives

Epilepsy is a common neurological disorder characterized by recurrent seizures. Epilepsy exhibits a complex etiology and pathogenesis involving abnormalities at multiple levels, including molecules, cells, and tissues. This review systematically explores the etiology and pathological mechanisms of epilepsy, with ion channel dysfunction, synaptic imbalance, immunity, neuroinflammation, metabolic dysregulation, and BBB disruption playing central roles. These mechanisms form a multifactorial regulatory network that collectively mediates the imbalance between excitatory and inhibitory signals in neurons. Further understanding the molecular pathogenesis of epilepsy provides an opportunity to discover new therapeutic targets and improve treatment accuracy. This review underscores recent advances in novel ASMs, minimally invasive surgery, neurostimulation, FUS, nanomedicine, GABAergic cell therapy, and gene therapy for the epilepsy management. These emerging strategies not only deepen our understanding of epilepsy treatment mechanisms but also hold considerable promise in both theoretical and clinical contexts. With improved efficacy and possibly fewer adverse effects compared to traditional ASMs and surgery, they suggest a transformative future for epilepsy therapies.

Although emerging therapies may bring better clinical outcomes, they still face several important challenges and limitations. Drug resistance and peripheral adverse effects remain significant limitations of novel ASMs [14]. Lower seizure freedom rates compared to open resection, as well as surgical risks, restrict the application of minimally invasive surgery [144]. Neurostimulation outcomes are often influenced by clinician preference, the absence of specific guidelines, and the low likelihood of achieving long‐term seizure freedom [15]. Despite the noninvasive nature of FUS, the risk of skull overheating remains a challenge that needs to be overcome. Nanomedicine is constrained by toxicology and biocompatibility concerns [394]. GABAergic cell therapy and gene therapy, although promising, remain largely underexplored due to high costs and limited clinical data. Reliance on rodent data is insufficient for clinical translation. Cell rejection poses a notable challenge in GABAergic cell therapy [395]. Delivery and specificity are issues that need to be addressed in gene therapy [392]. These factors make it difficult for some emerging therapies to achieve widespread clinical application in the short term. A summary of the advantages and limitations of these novel therapies is provided in Table 5.

**TABLE 5:** The advantages and limitations of the novel epilepsy therapy.

Treatment	Advantages	Limitations
Novel ASMs	Convenience and accessibility of treatment Low price Good compliance The first‐line therapy for all PWE Easy to manage and adjust treatment plans	Approximately 1/3 epilepsy patients have DRE Peripheral adverse effects Impairment of liver and kidney Palliative treatment
Minimally invasive surgery	Reduced invasiveness and lower morbidity Better preservation of cognitive function Shorter hospital stays and faster recovery High precision with robotic assistance	Lower seizure freedom rates compared to resection High initial costs and maintenance expenses Steep learning curve for surgeons Risk of complications like infection or bleeding
Neurostimulation	Continuous therapeutic effects Personalized Therapy Benefiting some DRE patients Improving quality of life	Invasive treatment Complications such as infection and bleeding Regular battery and electrode replacement is required Only applicable to some types of epilepsy Palliative treatment High cost
Nanomaterials	Accurately and comprehensively record epileptiform activity Nanomaterial‐enabled neuromodulation Nanocarriers facilitate ASMs transport across the BBB Assist in locating epileptic lesions	High cost Biological safety not confirmed Not yet regulated and standardized
FUS	Noninvasive treatment Ability to target deep tissue Precise positioning Real time monitoring during the treatment process Sublesion testing	The safety and effectiveness of FUS have yet to be firmly established Standardized parameters for FUS treatment are still unclear The possibility of increased skull heating
GABAergic cell therapy	Capacity to facilitate neural network reorganization Ability to treat multi‐lesional epilepsy Personalized Therapy	Lack of clinical evidence of human subjects The possibility of graft rejection High cost
Gene therapy	Minimal adverse effects Personalization Addressing unique genetic profiles Healing potential	High cost Time‐intensive nature The optimal treatment time window is unclear

Abbreviations: ASMs, anti‐seizure medications; BBB, blood–brain barrier; DRE, drug‐resistant epilepsy; FUS, focused ultrasound; PWE, patients with epilepsy.

Looking ahead, several critical avenues for future research and development are essential to overcoming current limitations and enhancing therapeutic efficacy. (1) Elucidating the core mechanisms of epilepsy, leveraging innovative platforms for therapeutic optimization, and establishing rapid translational pipelines constitute an integrative framework that bridges fundamental discovery with clinical validation. This approach promises to redefine therapeutic goals and outcomes for patients with DRE. Targeted therapies for circadian rhythm disorders and glymphatic dysfunction represent promising directions for future research [396, 397, 398]. (2) There is an urgent need to address the translational gap between animal models and human patients, as physiological differences between species often result in failures during clinical trials. Future research must prioritize the development of more human‐relevant models, such as brain organoids derived from hiPSCs [399]. These advanced models will provide more accurate information for assessing efficacy, optimizing dosing, and evaluating long‐term safety prior to human trials. (3) Technological innovation and standardization are critical components in advancing medical techniques. In the context of FUS, therapeutic outcomes are highly dependent on specific parameters. Therefore, future research must focus on establishing standardized and optimal parameters for sonication intensity, duration, and frequency to enhance efficacy while minimizing risks such as skull heating. The integration of AI for target identification and personalized treatment represents a significant advance in this field. (4) Enhancing the safety and biocompatibility of materials is essential, particularly in the realms of nanomedicine and implantable devices. Comprehensive investigations into the long‐term toxicity, biodistribution, and potential immune reactions associated with nanomaterials are imperative [266]. Research efforts should be directed toward designing biodegradable nanocarriers with improved targeting ligands to increase specificity and reduce off‐target effects. In the case of implanted electrodes used for neurostimulation, the development of next‐generation materials with superior biocompatibility is necessary to minimize inflammatory tissue responses and ensure long‐term functional stability [266]. (5) Combination therapies should be actively explored due to the intricate and multifactorial nature of epilepsy pathogenesis, which may render a singular therapeutic approach inadequate. Future research should focus on elucidating the synergistic effects of integrating various treatment modalities. For instance, employing FUS to transiently disrupt the BBB could enhance the delivery of nanocarrier‐encapsulated ASOs. (6) A paradigm shift toward personalized and precision medicine is imperative. The etiology and molecular mechanisms underlying epilepsy exhibit significant variability among patients, necessitating the identification of reliable biomarkers to predict treatment outcomes. This includes genetic profiling to tailor gene therapies to specific patients, advanced neuroimaging techniques for precise localization of the EZ for surgical intervention, and electrophysiological biomarkers to optimize neurostimulation strategies. The development of personalized treatment algorithms that integrate multi‐omics data will be crucial in aligning the most appropriate therapeutic interventions with individual patient profiles.

In conclusion, the management of epilepsy is transitioning from conventional symptom relief to targeting pathological mechanisms. Evidence from animal experiments and clinical trials clearly demonstrates the potential of these emerging therapies in epilepsy management. Future research requires integrated approaches combining basic science with clinical innovation to advance the clinical translation of more innovative therapeutic strategies. Ongoing investigation into the parameters of these therapies, as well as the development of supportive materials and equipment, will be important to optimizing their therapeutic efficacy. These efforts will facilitate the integration of these advanced strategies into biomedicine, ultimately improving epilepsy patient outcomes.

## Author Contributions

**Wanbin Huang**: writing – original draft. **Jiabin Zong**: writing – original draft. **Yu Zhang**: writing – review and editing. **Ming Li**: formal analysis. **Songqing Pan**: formal analysis. **Zheman Xiao**: conceptualization. All authors have read and approved the final manuscript.

## Funding

This research was supported by funding from the National Natural Science Foundation of China (82471577). Major and stubborn disease project of national administration of traditional Chinese medicine Interdisciplinary (ZDYN‐2024‐A‐094). Key Research and Development Program of Hubei province (JSCX202501515). Innovative Talents Foundation of Renmin Hospital at Wuhan University (JCRCYG‐2022‐006), Research on Degree and Graduate Education Teaching Reform at Wuhan University, Teaching Research Project of Wuhan University Medical Science Center (2024YB08).

## Ethics Statement

The authors have nothing to report.

## Conflicts of Interest

The authors declare no conflicts of interest.

## Data Availability

The authors have nothing to report.
